# Metal complexes featuring a quinazoline schiff base ligand and glycine: synthesis, characterization, DFT and molecular docking analyses revealing their potent antibacterial, anti-helicobacter pylori, and Anti-COVID-19 activities

**DOI:** 10.1186/s13065-024-01239-7

**Published:** 2024-08-10

**Authors:** M. S. A. Mansour, Abeer T. Abdelkarim, Ahmed A. El-Sherif, Walaa H. Mahmoud

**Affiliations:** Chemistry Department, Faculty of Science, Cairo University, Giza, 12613 Egypt

**Keywords:** Schiff base complexes, Spectral studies, Molecular docking, DFT, Antimicrobial activity, H-Pylori, Anti-COVID-19

## Abstract

Mixed ligand complexes of manganese(II), cobalt(II), copper(II), and cadmium(II)with an innovative Schiff base ligand denoted as (**L**_**1**_), 4-(2-((1E,2E)-1-(2-(p-tolyl)hydrazineylidene)propan-2-ylidene)hydrazineyl), served as the principal ligand, while glycine (L2) was employed as secondary ligand were successfully effectively characterized through a comprehensive set of analyses, including Elemental analysis, UV–Visible, FT-IR, Mass spectra, and conductometric measurements. Density functional theory (DFT) computations were executed to discern the enduring electronic arrangement, the energy gap, dipole moment and chemical hardness of the hybrid ligand assemblies. The proposed geometry for the complexes is a distorted octahedral structure. The antimicrobial efficacy of these compounds was assessed against a range of bacterial and fungal strains. Notably, these complexes exhibited promising antimicrobial activities, with the cadmium (II) complex demonstrating superior efficacy towards all tested organisms. These compounds were also examined for their antibiotic properties against *H. pylori* to explore their broader medical potential. The Schiff base ligand and its corresponding metal complexes displayed substantial potential as an antibiotic against *H. pylori*. Additionally, the antitumor potential of the synthesized complexes was assessed against MCF-7 (Breast carcinoma) cells—the **Cu (II)** complex demonstrated superior activity with the lowest IC_50_ value compared to cisplatin. Moreover, it exhibited reduced cytotoxicity towards normal cells (VERO cells) compared to cisplatin, establishing it as the most potent compound in the study. Furthermore, molecular docking was explored of the Schiff base ligand and its corresponding cadmium(II) complex. The analysis of the docking study yielded valuable structural insights that can be effectively utilized in conducting inhibition studies for example against COVID-19. This comprehensive study highlights these synthesized compounds' multifaceted applications and promising bioactive properties.

## Introduction

Utilizing organic ligands with heteroatoms as coordination sites has been a significant avenue in coordination chemistry, offering a diverse array of exciting and practical complexes. Ligands containing multiple donor heteroatoms have garnered global attention due to their robust chelating capability, enabling the formation of mono-, di-, or multinuclear complexes [1, 2]. Moreover, the active electron pair on these heteroatoms significantly contributes to the bonding in metal–ligand interactions. Schiff bases, distinguished by the existence of an azomethine moiety, are known for their ability to form chelating rings via coordination with transition metals. These compounds are crucial in synthesizing promising and potent anticancer drugs. Moreover, Schiff bases manifest a broad spectrum of biological functionalities encompassing antibacterial, antifungal, anti-inflammatory attributes, and antimalarial [2]. The biological efficacy of Schiff bases may be heightened through their complexation interaction with diverse transition metal ions [3, 4]. Schiff bases are favored ligands because they stabilize a broad spectrum of metals across various oxidation states. This capability enables precise control over the functionality of metals, contributing to a diverse array of valuable applications in industrial sectors. Additionally, their significant roles in organic synthesis further underscore their importance in the field [5–8]. Mn(II), Co(II), Cu(II), and Cd(II) Schiff bases have been specifically chosen for their potential biological activity due to their unique coordination chemistry and ability to interact with biological molecules due to their wide variety of donor atoms as N, O, and S [9–12]. Furthermore, Schiff bases and their complexes with transition metals are essential in various fields due to their unique structural properties and potential applications. Combining Schiff bases and transition metals offers potential applications in multiple fields, including catalysis, medicine, materials science, sensing, and environmental remediation [13, 14]. These complexes' unique structural features and versatility drive research efforts to explore their properties and develop new applications. As highlighted in previous studies, incorporating glycine as a secondary ligand can potentially influence the synthesized complexes' planarity, hydrophobicity, and coordination geometry [15]. Amino acids, characterized by the presence of -NH_2_ and -COOH functional groups, are well-recognized for their ability to form metal complexes, holding significant importance in biological and pharmaceutical contexts [16, 17]. Recently, there has been an increasing scholarly focus on complexes involving transition metals with amino acids due to their demonstrated efficacy as antibacterial and antifungal agents. These complexes have shown effectiveness against various Microorganisms like *Escherichia Coli* and *Staphylococcus aureus* and have potential applications in food and medical domains [18, 19]. *Helicobacter pylori*, a spiral-shaped Gram-negative microorganism, was first discovered in 1982 within the human gastric mucosa. Colonization by H. pylori within the gastric environment can result in various gastrointestinal issues, including persistent active gastritis and the presence of duodenal and gastric ulcers, alongside indigestion. Furthermore, it is associated with developing malignancies, including gastric mucosa-associated lymphoid tissue (MALT) B-cell lymphoma and gastric adenocarcinoma [20]. Apart from these gastric complications, H. pylori infection has been linked to systemic disorders such as idiopathic thrombocytopenic purpura, disruptions in iron metabolism leading to anemia, deficiencies in vitamin B_12_, coronary artery disease, neurodegenerative disorders, and gall bladder issues like as cholecystitis and the formation of gallstones [20]. By employing DFT calculations, researchers can predict various physicochemical properties and reactivity patterns, offering valuable insights into the behavior of metal complexes in different environments. Additionally, molecular docking studies play a crucial role in elucidating the interactions between metal complexes and biological targets, facilitating the exploration of their potential applications in medicinal chemistry, catalysis, and other fields [21, 22]. Integrating DFT and molecular docking approaches enables a comprehensive understanding of the structure–function relationships of metal complexes, thus paving the way for the rational design and optimization of novel materials and compounds with tailored properties and activities [21–25]. The objective of this research is to synthesize a Schiff base ligand (L_1_) via the condensation process of (E)-1-(2-(p-tolyl)hydrazineylidene)propan-2-one with 4-hydrazineylquinazoline and subsequently develop its corresponding complexes with manganese[II], cobalt[II], copper[II], and cadmium[II]. A comprehensive analysis utilizing various physico-chemical techniques was employed for characterization purposes. The study further examines the biological functionalities, encompassing antimicrobial, antifungal, and anticancer attributes of the Schiff base (**L**_**1**_) and its associated metal complexes [26]. Additionally, the investigation aims to assess the suppressive impacts of the Schiff base ligand (**L**_**1**_) and its corresponding metal complexes on H. pylori infection. Furthermore, computational molecular docking investigations were conducted to elucidate the potential interaction modes of the ligand and its Cd (II) complex with the active sites of various receptors (**3AHU, 1GHP, 2ZIC, 1NEK, 5JPE, 7DAE,** and **6W41**) [27, 28].

## Experimental section

### Materials and procedures

In this research, chemicals of high purity were utilized, including 4-hydrazineylquinazoline 98% and (E)-1-(2-(p-tolyl)hydrazineylidene)propan-2-one 99% (sourced from Merck), glycine ≥ 99% and Manganese(II) chloride tetrahydrate ≥ 99% (MnCl_2_.4H_2_O) (obtained from Sigma-Aldrich), Cobalt(II) chloride hexahydrate 98%(CoCl_2_.6H_2_O), Copper chloride dehydrate 99% (CuCl_2_.2H_2_O) and Cadmium chloride hydrate 99.99%(CdCl_2_.2H_2_O), (procured from BDH). Absolute Ethanol (Ethyl alcohol 99,9%), an organic solvent of spectroscopic purity, was sourced from BDH. For all preparations, bidistilled water obtained from glass equipment was consistently used. The human tumor cell line, MCF-7, stored in liquid nitrogen at −180 °C, was obtained from the American Type Culture Collection. Cultivation and maintenance of the MCF-7 tumor cell line were conducted at the National Cancer Institute in Cairo, Egypt, through serial sub-culturing.

#### Solutions

Reservoir solutions containing the Schiff base ligand (**L**_**1**_), glycine and their corresponding metal complexes, each formulated at a specific concentration of 1 X 10^−3^ M, were created by precisely dissolving the appropriate weight in N, N‐dimethylformamide for Cu(II) complex and in ethanol for Schiff base ligand (**L**_**1**_), glycine (**L**_**2**_), Mn(II), Co(II), and Cd(II) complexes. The conductivity of the 1 X 10^−3^ M metal complex solution was then measured. Subsequently, solutions of the Schiff base ligand and its ternary metal complexes were diluted to a concentration of 1 X 10^−4^ M, utilizing precise dilution techniques from the initially prepared reservoir solutions for UV–Vis spectra measurement.

#### Solution of anti-*H. Pylori* study

##### Preparation of microbial suspensions

An initial culture of each microbial strain employed in susceptibility assessments was generated by transferring recent colonies of the microorganisms into tubes filled with sterile physiological sodium chloride solution. The turbidity of the inoculum was then adjusted to the 2.0 McFarland standard. This specific turbidity level produces a suspension density equivalent to (1.0 × 10^8^) CFU/mL of *H. pylori* [29].

##### Assessment of inhibitory effects against H. pylori

The well agar diffusion method assessed the in vitro anti-*Helicobacter pylori* (*H. pylori*) activities. In brief, a 100 μL volume of an H. pylori suspension ((1.0 × 10^8^) colony-forming units (CFUs)/mL) was evenly spread onto Mueller Hinton agar plates (BBL) supplemented with 10% sheep blood. Subsequently, a well with a diameter ranging from 6 to 8 mm was created using a sterile cork borer or tip. A 100 μL volume of the antimicrobial agent at the specified concentration was introduced into the well. Dimethyl sulfoxide (DMSO) served as the (−ve) control, while the antibiotics amoxicillin (AMX, (0.05) mg/mL), clarithromycin (CLR, (0.05) mg/mL), and metronidazole (MTZ, (0.8) mg/mL) were employed as (+ ve) controls. Following 72 h of incubation at 37 °C in a microaerophilic environment with humidity, the diameter of the inhibition zone was measured [30].

#### Solution for anticancer investigation

A freshly prepared reservoir solution (1 × 10^−3^ M) of the tested compounds using ethanol (90%) in an appropriate volume. For cell cryopreservation, DMSO was employed, while the RPMI‐1640 medium, formulated for the cultivation and sustenance of a human tumor cell line, was synthesized from its powdered state. The procedure entailed the amalgamation of 10.40 g of a specified medium with 2 g of sodium bicarbonate, finalizing the composition to a volume of 1 L by incorporating distilled water and promoting dissolution through meticulous agitation. Following this, the medium underwent sterilization through filtration using a Millipore bacterial filter with a pore size of 0.22 µm, after which it was stored at a temperature of 4 degrees Celsius. Regular assessments were conducted to monitor and detect any signs of contamination. The RPMI-1640 medium was preconditioned to 37 °C in a water bath before application, and it underwent supplementation with penicillin–streptomycin and fetal bovine serum (FBS). The inclusion of sodium bicarbonate was imperative in the formulation of the RPMI-1640 medium. A 0.05% isotonic trypan blue solution was prepared using normal saline for assessing cell viability. Before use, the RPMI-1640 medium was fortified with 10% heat-inactivated FBS (at 56 °C for 30 min), 100 units/ml of penicillin, and 2 mg/mL of streptomycin. Cell harvesting employed trypsin (0.25 X 10^−1^% w/v), while unbound sulforhodamine B (SRB) dye was dissolved using acetic acid (1% v/v). The protein–dye SRB (0.40%) was dissolved in 1% acetic acid for its intended application. A stock solution containing trichloroacetic acid (TCA) at a concentration of 50% was prepared and stored for subsequent use. The experimental protocol involved adding 50 μL of the prepared stock solution to 200 μL of RPMI-1640 medium per well, resulting in a final concentration of 10% TCA for protein precipitation. Additionally, 100% isopropanol and 70% ethanol were employed in the experimental procedures. For the solubilization of sulforhodamine B (SRB) dye, Tris base at a concentration of 10 mM and a pH of 10.50 was utilized. Specifically, 121.10 g of Tris base was dissolved in 1000 mL of distilled water, and the pH was adjusted to the desired level using hydrochloric acid with a concentration of 2 M.

#### Instrumentation

The elemental composition analysis of carbon, hydrogen, and nitrogen was executed at Cairo University's Microanalytical Center in Egypt, utilizing a CHNS-932 (LECO) Vario elemental analyzer. Melting point determinations were conducted with the triforce XMTD-3000 instrument. Metal content analyses were performed at the Egyptian Petroleum Research Institute utilizing inductively coupled plasma spectrometry (ICP). Fourier-transform infrared spectroscopy (FT-IR) analyses, spanning from 4000 to 400 cm^−1^, were recorded using KBr disks and a Perkin-Elmer 1650 spectrometer. The molar conductivities of 10^–3^ M solutions of the solid complexes in DMF were determined using a Jenway 4010 conductivity meter. For solutions in DMSO-*d6*, ^1^H NMR spectra were obtained with tetramethylsilane as an internal standard, utilizing a 300 MHz Varian-Oxford Mercury instrument at room temperature. Mass spectra were acquired through the electron ionization technique at 70 eV employing an MS-5988 GS-MS Hewlett-Packard instrument at Cairo University's Microanalytical Center. Thermal analyses, including thermogravimetric (TG) and differential thermogravimetric (DTG) measurements of the Schiff base ligand and its ternary metal complexes, were conducted using a Shimadzu TG-50H thermal analyzer, spanning the temperature range from room temperature to 1000 °C. Solution-based spectrophotometric measurements were carried out using an automated UV–Vis spectrophotometer, PerkinElmer Model Lambda 20, covering the wavelength range from 200 to 700 nm. Anticancer activity experiments were executed at the National Cancer Institute, specifically within the Cancer Biology and Pharmacology Departments at Cairo University, Egypt. Antimicrobial assessments were performed at Cairo University's Microanalytical Center. Anti-H. Pylori activity assessments were conducted at the Microbiology Center at Alazhar University.

### Preparation of Schiff base ligand

**Scheme 1:**
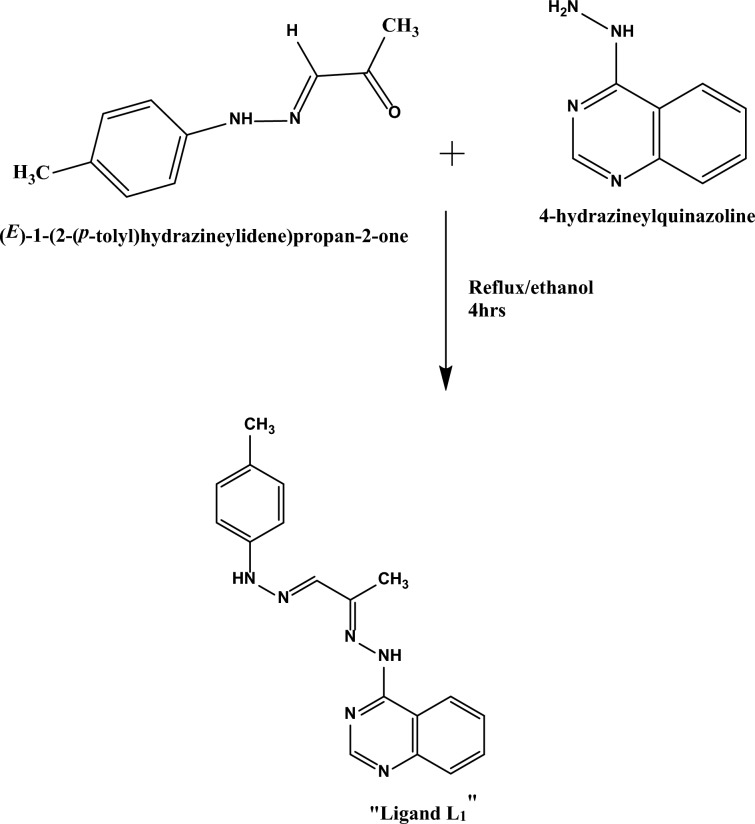
Synthetic route for Schiff Base ligand (**L**_**1**_)

Continuing our previous work synthesizing new Schiff base Complexes [13, 31, 32], the recently developed Schiff base ligand (L_1_) was synthesized using a standard method. The process involved the condensation of 4-hydrazineylquinazoline (4.370 mmol, 0.7 g) and (E)-1-(2-(p-tolyl)hydrazineylidene)propan-2-one (4.370 mmol, 0.770 g) in hot 100% ethanol at 60 °C in a round-bottom flask. The reaction mixture underwent reflux for 4 h in an open system under controlled laboratory conditions to ensure thorough mixing. During reflux, the reaction progressed, leading to the formation of the desired Schiff base ligand. After the reflux time, the reaction mixture was allowed to cool to room temperature. The resulting pure Schiff base was obtained with a yield of 96%, filtering and recrystallizing the resultant orange solid. Scheme 1 illustrates the configuration of the Schiff base ligand **(L**_**1**_**)** and the formation reaction involved in its creation. Yield 96%; orange solid, m.p 210 °C. The theoretical composition for C_19_H_19_N_5_ is anticipated to be as follows: Carbon (C) 71.90%, Hydrogen (H) 6.03%, and Nitrogen (N) 22.07%. The experimental findings yield the following actual composition: Carbon (C) 71.53%, Hydrogen (H) 5.88%, and Nitrogen (N) 21.82%. IR (ν, cm^−1^): 3437sh (NH), 1605 sh (C = N), 1545sh (υ(C = N) Pyridine. In the ultraviolet–visible (UV–Vis) spectroscopic analysis, distinct absorption peaks were observed at wavelengths of 314 (attributed to π–π* transitions), 334 (indicative of n–π* transitions), and 388(associated with charge transfer).

### Preparation of ternary complexes

**Scheme 2:**
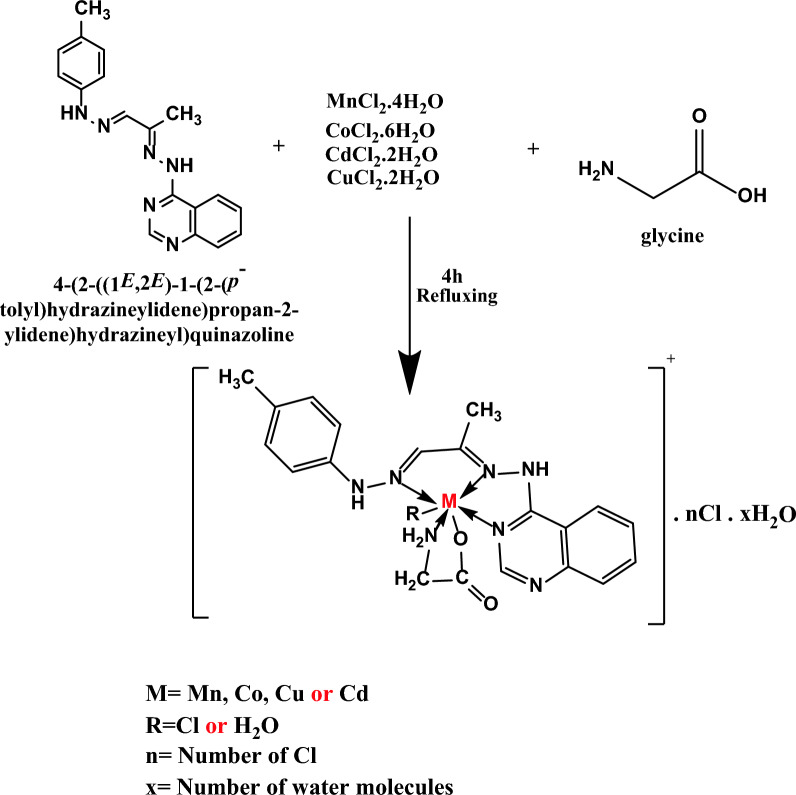
Structure of the mixed ligands complexes and their formation reaction

For synthesizing the mixed ligand complexes, the synthesized Schiff base ligand serves as the principal ligand denoted as (**L**_**1**_). The Schiff base ligand (**L**_**1**_) was formulated by dissolving (0.314 mmol, 0.10 g) in 40 ml of heated absolute ethanol. The secondary ligand (**L**_**2**_), the amino acid Glycine (0.314 mmol, 0.02 g), was solubilized in a 10-ml volume of heated distilled water. The chelates were formed by combining a hot solution of the primary Schiff base ligand (L_1_) in absolute ethanol with the secondary ligand glycine in distilled water. To this mixture, a hot ethanoic solution (20 ml) of the relevant metal chloride salt (such as MnCl_2_.4H_2_O, CoCl_2_ 0.6H_2_O, CuCl_2_.2H_2_O, and CdCl_2_.2H_2_O,) (0.314 mmol) was gradually added dropwise. The resultant solution underwent reflux for three hours, leading to the formation of precipitates representing the complexes. These precipitates were collected via filtration and further purified through multiple washes using a combination of ethanol and water. Subsequently, they underwent desiccation within a vacuum environment employing anhydrous CaCl_2_. Scheme 2 illustrates the structure configuration of the mixed ligands complexes and their formation reaction.

#### [Mn(L_1_)(L_2_)(H_2_O)].Cl.H_2_O

Yield 96%; m.p. = 274 °C; Brown solid. Anal. Calc. for (**C**_**20**_**H**_**26**_**ClMnN**_**7**_**O**_**4**_) (%): C, 46.25; H, 5.01; N, 18.90; Cl 6.83; Mn, 10.58 Found (%): C, 46.08; H, 4.88; N, 18.77; Cl 6.72; Mn, 10.46. Λm (Ω^−1^ mol^−1^ cm^2^) = 86; FT‐IR (ν, cm^−1^): Azomethine (C = N) 1610, azomethine (C = N)_Pyridine_ 1560 m, ν (COO)asy 1421 m, ν (COO)sym 1336 s, (M─O) 506w, (M─N) 460w. UV–Vis (λmax, nm): 307 (π–π*), 310 (π–π*) conjugated, 369(n- π*).

#### [Co(L_1_)(L_2_)(H_2_O)].Cl.H_2_O

Yield 97%; m.p. = 285 °C; dark brown solid. Anal. Calc. for (**C**_**20**_**H**_**26**_**ClCoN**_**7**_**O**_**4**_) (%): C, 45.94; H, 4.97; N, 18.75; Cl 6.78; Co, 11.27 Found (%): C, 45.87; H, 4.88; N, 18.51; Cl 6.62; Co, 11.17. Λm (Ω^−1^ mol^−1^ cm^2^) = 79; FT‐IR (ν, cm^−1^): Azomethine (C = N) 1619, azomethine (C = N)_Pyridine_ 1563 m, ν (COO)asy 1410 m, ν (COO)sym 1324 s, (M─O) 547w, (M─N) 458w. UV–Vis (λmax, nm): 316 (π–π*), 347(n- π*).

#### [Cu(L_1_)(L_2_)(H_2_O)].Cl.2H_2_O

Yield 96%; m.p. = 292 °C; Reddish brown solid. Anal. Calc. for (**C**_**20**_**H**_**28**_**ClCuN**_**7**_**O**_**5**_) (%): C, 44.03; H, 5.13; N, 17.97; Cl 6.49; Cu, 11.65 Found (%): C, 43.95; H, 5.03; N, 17.85; Cl 6.38; Cu, 11.51. Λm (Ω^−1^ mol^−1^ cm^2^) = 82; FT‐IR (ν, cm^−1^): Azomethine (C = N) 1628, azomethine (C = N)_Pyridine_ 1559 m, ν (COO)asy 1413 m, ν (COO)sym 1327 s, (M─O) 560w, (M─N) 436w. UV–Vis (λmax, nm): 292 (π–π*), 362(n- π*), 360 (Charge transfer).

#### [Cd(L_1_)(L_2_)Cl]

Yield 97%; m.p. = 261 °C; yellow solid. Anal. Calc. for (**C**_**20**_**H**_**22**_**CdClN**_**7**_**O**_**2**_) (%): C, 44.45; H, 4.07; N, 18.51; O, 5.92; Cl 6.56; Cd, 20.80 Found (%): C, 44.21; H, 4.00; N, 18.06; O, 5.85; Cl 5.42; Cd, 20.66. Λm (Ω^−1^ mol^−1^ cm^2^) = 16; FT‐IR (ν, cm^−1^): Azomethine (C = N) 1620, azomethine (C = N)_Pyridine_ 1573 m, ν (COO)asy 1422 m, ν (COO)sym 1336 s, (M─O) 586w, (M─N) 469w. UV–Vis (λmax, nm): 280 (π–π*), 315 (π–π*) conjugated, 360(n- π*).

### Exploring spectrophotometry

Absorption spectra were recorded for solutions containing the free Schiff base ligand and its ternary corresponding metal complexes at a concentration of (1 × 10^−4^ M). The spectra were systematically scanned over the wavelength range from 200 to 700 nm.

### Computational methodology

Theoretical calculations were conducted on the free Schiff base ligand (**L**_**1**_) and its corresponding metal complexes utilizing the software package (Gaussian 09w). Density functional theory (DFT) served as the chosen level of theory for these calculations. The structural configuration geometry of the ligand was extensively optimized through the utilization of the B3LYP method in combination with (LANL2DZ) basis set for Schiff base ligand (**L**_**1**_) and its corresponding metal complexes [33]. The Chemcraft version 1.8 software suite and Gaussian View version 6.0.1 package were employed to visualize the optimized structure of the ternary complex. Key chemical quantum parameters, including (E_HOMO_) and (E_LUMO_), as well as the HOMO–LUMO energy gap (ΔE), were computed for the studied molecule. These parameters offer crucial information regarding the electronic configuration and reactivity of the ligand.

The B3LYP method, in combination with the LANL2DZ basis set, is commonly used in computational chemistry calculations for several reasons [34, 35]:

Accuracy and reliability: The B3LYP functional, which is a hybrid functional that incorporates a mixture of Hartree–Fock exchange and density functional exchange–correlation, has been extensively tested and proven to provide accurate results for a wide range of systems, especially for molecular geometries, vibrational frequencies, and thermochemical properties [36, 37].

Balanced performance: The B3LYP functional balances computational cost and accuracy, making it a popular choice for moderately sized systems and transition metal complexes, where more computationally demanding methods may not be feasible. Transition metal complexes: The LANL2DZ basis set is handy for transition metal systems. It is an effective core potential (ECP) basis set, which incorporates relativistic effects and treats the core electrons implicitly, reducing the computational cost while providing reasonable accuracy for transition metal complexes [38, 39].

Benchmark studies: Numerous benchmark studies have been conducted on the performance of the B3LYP/LANL2DZ combination for various properties of transition metal complexes, providing a reference point and validation for its use in specific applications [40, 41].

### Antimicrobial activity

The investigation into the antimicrobial efficacy of the examined compounds utilized the agar well diffusion method. In vitro assays were performed to evaluate antibacterial properties against Gram (+ ve) bacteria, specifically *Staphylococcus aureus* and *Streptococcus mutans,* as well as Gram (-ve) bacteria, including *Escherichia coli*, *Pseudomonas aeruginosa*, and *Klebsiella pneumonia*. These assessments were conducted on a nutrient agar medium. Antifungal activity was appraised towards *Candida albicans* and *Aspergillus niger* using Sabouraud dextrose agar medium. Standard drugs, including (Gentamicin and Ampicillin) for Gram (−ve) and Gram (+ ve) bacteria, respectively, and Nystatin for fungal strains, were employed as reference points. Dimethyl sulfoxide (DMSO) served as the control (negative) solvent. The compound concentration tested against bacterial and fungal strains was 15 mg/mL.

#### Testing method

The aseptic culture medium was dispensed into Petri dishes, each receiving an approximate volume of 20–25 mL, and subsequently allowed to solidify at ambient temperature. A microbial suspension, mirroring the McFarland 0.5 standard solution (1.5 × 105 CFU mL^−1^), was meticulously prepared in sterilized saline. The suspension's turbidity was precisely adjusted to an optical density (OD) of 0.13 using a spectrophotometer set at 625 nm [30]. Within a time frame of approximately 15 min post-turbidity adjustment, a sterile cotton swab was immersed in the prepared microbial suspension and uniformly streaked onto the desiccated agar surface. The swab-laden dish was left undisturbed for 15 min with the lid securely in place. Subsequently, employing a sterile borer, wells measuring 6 mm in diameter were generated in the solidified culture medium [30]. Then, 100 μL of the test compound solution was carefully added to each well using a micropipette. The specimens were subsequently incubated at 37 degrees Celsius for 24 h to observe antibacterial activity. This experimental process was conducted in triplicate, and the diameters of the inhibition zones were assessed in millimeters, as reported in a prior study [30].

### Anti-H. Pylori activity

The assessment of Schiff base (**L**_**1**_) and its metal complexes' efficacy against *H. pylori* ATCC 43504 involved the application of the micro-dilution broth method. The determination of minimal inhibitory concentration (MIC) and minimal bactericidal concentration (MBC) for the tested compounds was carried out using Mueller–Hinton broth supplemented with lysed horse blood [42].

#### Minimal inhibitory concentration (MIC)

The micro-dilution broth method determined the test compounds' minimal inhibitory concentration (MIC). This method involved the use of Mueller–Hinton broth supplemented with lysed horse blood. Serial two-fold dilutions were systematically performed to establish a concentration range from 0.98 to 1000 μg/mL for the tested extracts. Sterile 96-well polystyrene microtitrate plates were employed, and each well was loaded with 200 μL of the respective extract dilution in the broth medium. Inocula were prepared by cultivating fresh microbial cultures in sterile 0.85% NaCl, with adjustments made to achieve turbidity matching the 1.0 McFarland standard. Subsequently, 2 µL of the prepared inoculum was added to each well, resulting in a final density of 3.0 × 106 CFU (colony-forming units)/mL. Following an incubation period of 72 h at 35 °C under microaerophilic conditions (15% CO_2_), the MICs were determined by visually identifying the lowest extract concentration that completely inhibited the growth of the reference strain. Every microplate comprised a positive control, wherein inoculum was present without the test extracts, and a negative control, wherein the test extracts were present without inoculum [42].

#### Minimal bactericidal concentration (MBC)

The Minimum Bactericidal Concentration (MBC) was ascertained by inoculating 100 mL of the microbial culture derived from wells displaying complete growth inhibition, observed in both the last (+ ve) and growth control wells, onto Mueller–Hinton agar plates supplemented with 5% horse blood. Subsequently, the plates underwent incubation at 35 °C for 72 h under a microaerophilic environment. The MBC was defined as the lowest concentration of the extracts where no microbial growth was observed. To ascertain the bactericidal or bacteriostatic effects of the tested extracts, MBC/MIC ratios were calculated. Notably, in this study, antibacterial agents were considered bactericidal if the MBC/MIC ratio did not exceed four times the MIC [43]. Each trial was executed in triplicate, and the presented data represent the findings.

### Anticancer activity

To evaluate the cytotoxic effects of both the Schiff base ligand (**L**_**1**_) and its associated metal complexes, the study followed a methodology developed by Skehan and Storeng [44]. This method involved utilizing a 96-well plate with 104 cells per well, allowing 24 h to facilitate cell adhesion to the well's surface. The compounds under investigation, including the Schiff base ligand and its metal complexes, underwent examination across diverse concentrations (100, 50, 25, and 12.5 μg mL^−1^). with each concentration assessed in triplicate. After introducing the test compounds, the cellular monolayer underwent a 48-h incubation period at 37 °C within an atmosphere containing 5% CO_2_. After this incubation, the cells were subjected to fixation, thorough washing, and SRB stain staining. Surplus stain was eliminated through treatment with acetic acid, and the residual staining was extracted using a tris–EDTA buffer solution. The optical density (O.D.) of individual well samples was assessed at a wavelength of 564 nm employing a spectrophotometer, with automatic subtraction of the mean background absorbance. Mean values for each concentration of the tested compounds were subsequently calculated. To assess the impact of the compounds on cell survival, a relation was established between the surviving fraction and the drug concentration, generating a survival curve for the specific breast tumor cell line being studied. The percentage of cell survival was determined using the following formula:

$${\mathbf{Survival}} \, {\mathbf{fraction}} \, = \, {\mathbf{O}}.{\mathbf{D}}. \, {\mathbf{of}} \, {\mathbf{treated}} \, {\mathbf{cells}}/{\mathbf{O}}.{\mathbf{D}}. \, {\mathbf{of}} \, {\mathbf{control}} \, {\mathbf{cells}}.$$

The primary goal of this study was to calculate the IC_50_ values, which represent the concentrations of the free Schiff base ligand (**L**_**1**_) or its associated metal complexes needed to inhibit cell growth by 50%. The entire experiment was meticulously repeated three times for the MCF7 cell line to ensure the reliability and consistency of the results.

### Molecular docking

Molecular docking analyses were conducted utilizing the MOE 2014 software, which is recognized for its rigid molecular docking capabilities. These analyses were pivotal in predicting potential binding modes of both the Schiff base ligand (**L**_**1**_) and its Cadmium [II] complex with significant receptors. Specifically, the study targeted crystal structures of essential receptors for bacterial and fungal strains, including (PDB ID: **3AHU, 1GHP, 2ZIC, 1NEK, 5JPE**), in addition to microtubules, crucial elements of the cytoskeleton associated with cancer treatment (PDB ID: **7DAE**), and (PDB ID: **6W41**) the crystal structure of the SARS-CoV-2 receptor binding domain bound to the human antibody (CR3022). It can compute and present likely docking configurations of the receptors with the Schiff base ligand and its complex molecules. The software necessitates input in PDB format, comprising the Schiff base ligand, the ternary cadmium [II] complex, and the receptors. While preparing the input files, extraneous components such as aqueous entities, co-crystallized ligands, and unaccompanied elements such as sodium, potassium, mercury, etc., should be excluded. However, the amino acid chains were retained for further analysis [45]. The Schiff base ligand and its ternary Cadmium [II] complex were generated in PDB file format using Gaussian software. The structural configurations of each receptor were acquired from the Protein Data Bank (PDB), accessible at http://www.rcsb.org/pdb. This methodology allows for investigating potential binding interactions and evaluating the interplay of the Schiff base ligand (**L**_**1**_) and its Cadmium [II] complex with these vital receptors. This analysis provides valuable insights into possible therapeutic applications.

## Results and discussion

### Characterization of the Schiff base (L_1_)

**Fig. 1:**
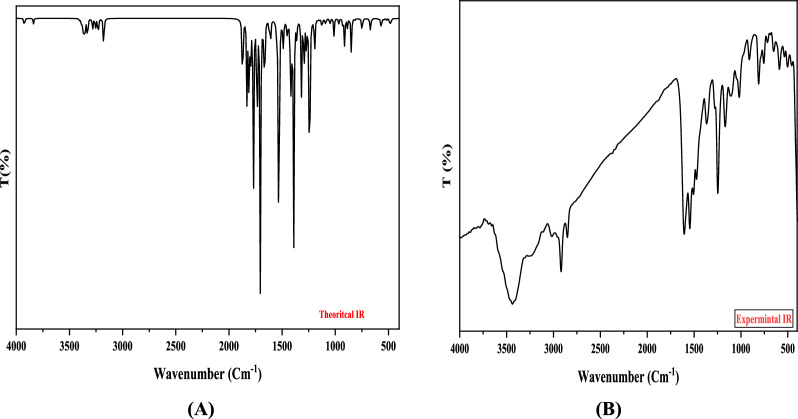
Infrared spectroscopy analysis of Schiff Base ligand **(L**_**1**_**)**: **A** theoretical spectrum and **B** experimental spectrum

**Fig. 2:**
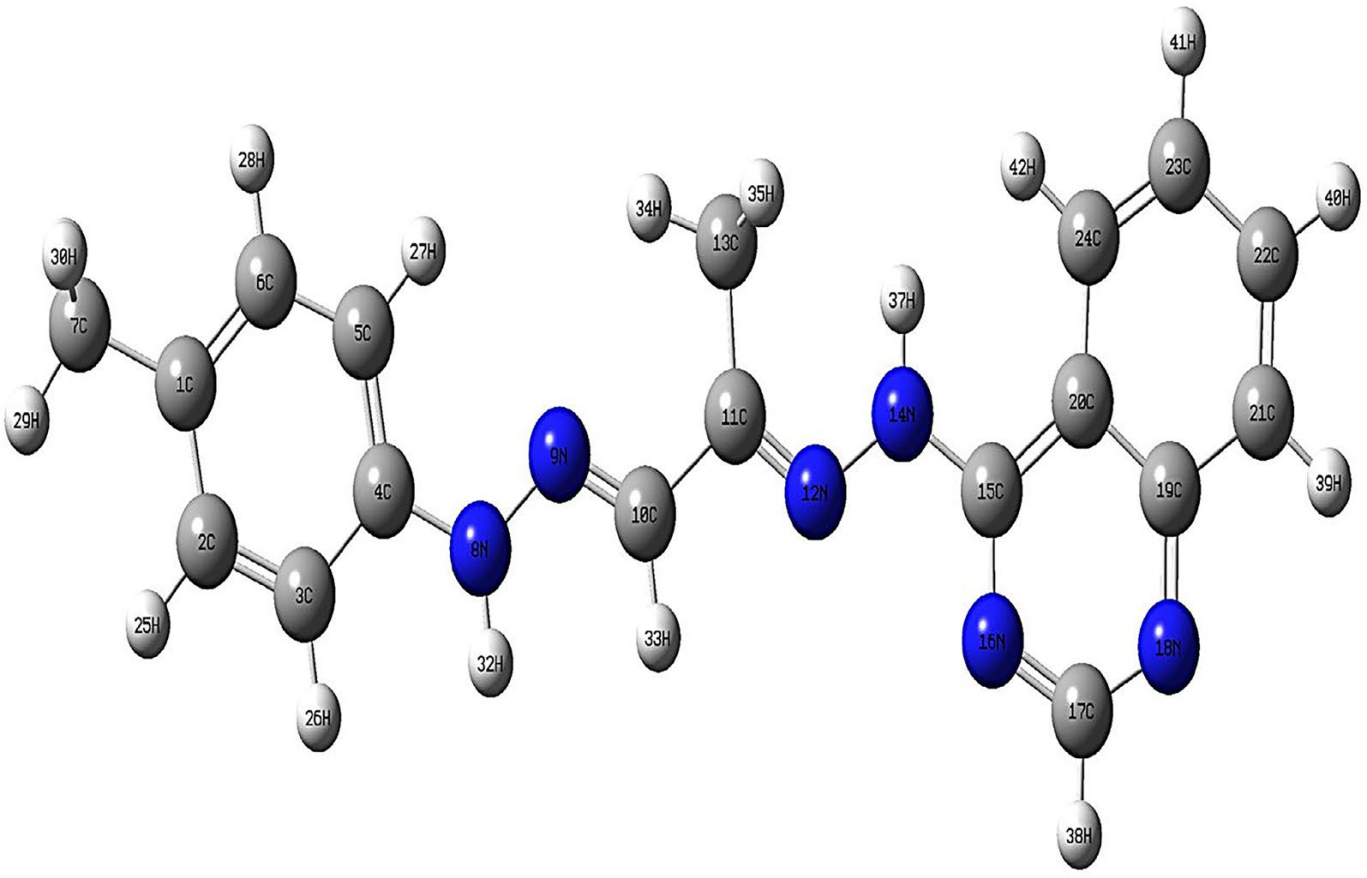
The optimized configuration structure of Schiff base ligand (**L**_**1**_)

The elemental analysis of the tridentate Schiff base ligand (**L**_**1**_) revealed experimental data consistent with the calculated values, affirming its molecular formula as **C**_**18**_**H**_**18**_**N**_**6**_. This compound exhibited favorable physical properties: it had a melting point of approximately 210 °C and demonstrated solubility in various solvents such as DMF and DMSO, displaying an orange coloration. The proton of the NH group was detected in the Schiff base ligand's ^1^H NMR spectrum at 9.68 and 11.13 ppm, along with azomethine protons at 8.49 ppm and aromatic protons between 7.45 and 8.22 ppm (see Table 3). The lack of observable indications of any evidence for the NH2 group confirms the synthesis of the Schiff base ligand (**L**_**1**_). Further structural insights into Schiff base ligand (**L**_**1**_) were obtained through its mass spectrum analysis. The recorded spectrum depicted a molecular ion peak at m/z = 318.78 a.u, consistent with the ligand's formula weight of **C**_**18**_**H**_**18**_**N**, having a molecular weight of 318.38 g/mol. In the UV–Vis absorption spectrum of the Schiff base ligand (**L**_**1**_), three discernible bands manifested at 314 nm, indicative of π‐π* transitions, at 334 nm, associated with n‐π* transitions, and at 388 nm, correlated with charge transfer phenomena. Significant bands corresponding to the ligand's structure were observed in the prepared Schiff base ligand's infrared spectrum. Notably, a notable band at 1605 cm^−1^ signified the existence of two azomethine groups, while the band spectral feature at 1545 cm^−1^ corresponded to (C = N) Pyridine, as listed in Table 5. A broad vibration band attributed to ν (NH) was also detected at 3437 cm^−1^ [46]. These bands were recorded by DFT at 3580, 1655, and 1590 cm^−1^, respectively. The concurrence of the theoretical and experimental frequencies was perfect and indicated the successful preparation of the Schiff base ligand and supported the chemical structure of the ligand, as illustrated in Fig. 1. The differences between theoretical and experimental frequencies may result from systematic errors (derived from harmonicity) or the use of gas phase molecules in the DFT calculations. So, the correlation coefficient was used as 0.9648 for LAN2DZ to overcome these errors [33, 47]. In these investigations, Fig. 2 elucidates the molecular configuration of the Schiff base ligand denoted as **L**_**1**_.

### Characterization of Schiff base ternary metal complexes

#### Elemental analysis

Four metal complexes were synthesized via the combination of equimolar quantities of a Schiff base (**L**_**1**_) and glycine ligand (**L**_**2**_) with manganese(II), cobalt(II), copper(II), and cadmium(II) metal salts in suitable solvents. Detailed elemental analyses and an exploration of these compounds' molecular formulae, melting points, and various physical properties were conducted. These complexes demonstrated stability at room temperature and exhibited high sharp melting points. They were determined to exhibit limited solubility in water and high solubility in DMF and DMSO solvents. The estimated values and the experimental elemental analysis of the complexes demonstrated a remarkable level of agreement. As indicated in Table 1, the complexes prominently manifest a **1:1:1** ratio, wherein a single metal ion forms a bond with one Schiff base (**L**_**1**_) ligand and one glycine ligand. Consequently, this outcome gives rise to a complex with stoichiometry denoted as **M: L**_**1**_**: L**_**2**_**.**

**Table 1:** Displays the physical and analytical data concerning the Schiff base ligand (**L**_**1**_) and its associated ternary metal complexes

Compound "chemical formula"	Color (%)(Yield)	M.p. (°C)	Found (Calcd)					A_m_ (Ω^−1^ mol^−1^ cm^2^)
			C (%)	H (%)	N (%)	Cl (%)	M (%)	
Schiff base ligand (**L**_**1**_) **C**_**19**_**H**_**19**_**N**_**5**_	Orange (96)	210	71.53 (71.90)	5.88 (6.03)	21.82 (22.07)			
**[Mn(L**_**1**_**)(L**_**2**_**)(H**_**2**_**O)].Cl.H**_**2**_**O** **(C**_**20**_**H**_**26**_**MnClN**_**7**_**O**_**4**_**)**	Dark brown (97)	285	45.87 (45.94)	4.88 (4.97)	18.51 (18.75)	6.62 (6.78)	11.17 (11.27)	86
**[Co(L**_**1**_**)(L**_**2**_**)(H**_**2**_**O)].Cl.H**_**2**_**O** **(C**_**20**_** H**_**26**_**CoClN**_**7**_**O**_**4**_**)**	yellow (97)	261	44.21 (44.45)	4.00 (4.07)	18.06 (18.51)	5.42 (6.56)	20.66 (20.80)	79
**[Cu(L**_**1**_**)(L**_**2**_**)(H**_**2**_**O)].Cl.2H**_**2**_**O** **(C**_**20**_** H**_**28**_**CuClN**_**7**_**O**_**5**_**)**	Brown (96)	274	46.08 (46.25)	4.88 (5.01)	18.77 (18.90)	6.72 (6.83)	10.46 (10.58)	82
**[Cd(L**_**1**_**)(L**_**2**_**)Cl]** **(C**_**20**_**H**_**22**_**CdClN**_**7**_**O**_**2**_**)**	Reddish brown (96)	292	43.95 (44.03)	5.03 (5.13)	17.85 (17.97)	6.38 (6.49)	11.51 (11.65)	16

#### EDX-SEM analysis

**Fig. 3:**
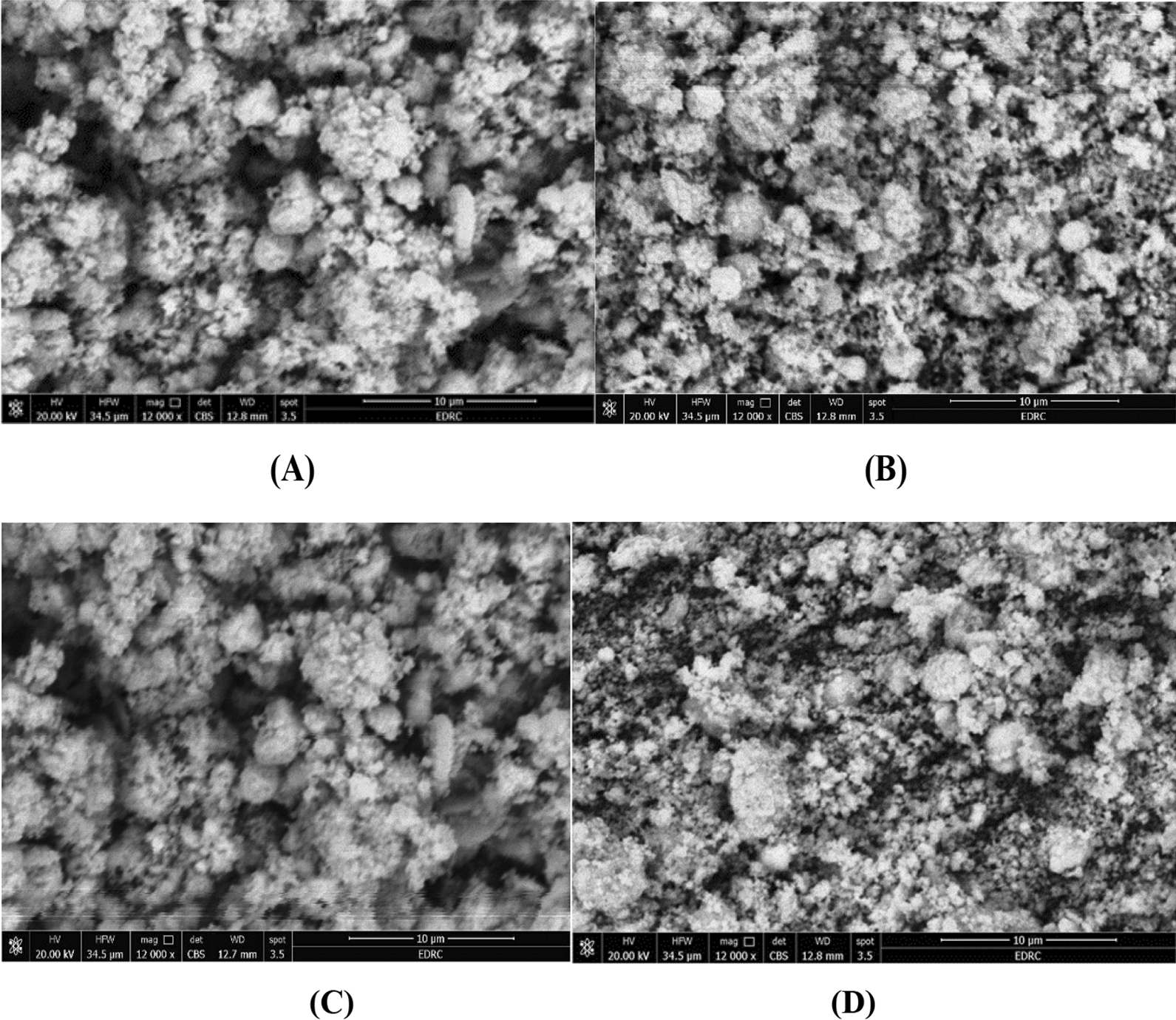
Morphological analysis of Schiff Base metal complexes via SEM [**A** Mn(II), **B** Co(II), **C** Cu(II), **D** Cd(II)]

SEM and EDX analyses were performed to investigate Schiff base complexes' morphology and elemental composition [48]. the Mn(II) complex exhibited a morphology in the SEM image Characterized by irregular, elongated structures. The individual structures have varying lengths and diameters, suggesting a non-uniform growth process (Fig. 3A). The EDX analysis (Table S4) revealed manganese complex at 11.17% (calculated 11.27%), matching well with the elemental analysis of manganese at 11.17% (calculated 11.27%), indicating successful formation of the expected **C**_**20**_**H**_**26**_**MnClN**_**7**_**O**_**4**_ composition. For the Co(II) complex, the SEM image characterized by overlapping sheet-like structures. These sheet-like structures appear to be stacked and intertwined, creating a layered arrangement. The sheets seem to have smooth surfaces with well-defined edges, indicating a more ordered growth process compared to the Mn(II) complex (Fig. 3B). The EDX data (Table S2) aligned with the elemental composition analysis (Table 1), detecting cobalt (20.66% found vs 20.80% calculated) and other elements consistent with the **C**_**20**_**H**_**26**_**CoClN**_**7**_**O**_**4**_ composition. The Cu(II) complex displayed a different morphological pattern in the SEM micrograph reveals a different morphological pattern from the Mn(II) and Co(II) complexes. The Cu(II) complex exhibits a structure composed of irregularly shaped particles or agglomerates. These particles appear to be randomly oriented and loosely packed, with a rough surface texture. The lack of a well-defined shape or ordered arrangement suggests a more disordered growth process (Fig. 3C). The EDX data (Table S3) Correlated with the elemental composition analysis of copper at 10.46% (calculated 10.58%) (Table 1) showed copper along with C, N, O indicating successful formation of the **C**_**20**_**H**_**28**_**CuClN**_**7**_**O**_**5**_ complex. Finally, The Cd(II) Schiff base complex exhibited exhibits a well-defined crystalline structure, as evident from the SEM image. The crystals appear to be cubic or rectangular in shape, with distinct edges and smooth faces. These crystals are agglomerated, forming larger clusters or crystalline assemblies. The regular shape and well-defined facets of the crystals indicate a highly ordered growth process for the Cd(II) complex (Fig. 3D). This morphology correlated well with the elemental composition data which showed the presence of cadmium (11.51% found vs 11.65% calculated) along with carbon, nitrogen, oxygen (Table 1) in agreement with data from EDX analysis (Table S1), confirming the formation of the expected **C**_**20**_**H**_**22**_**CdClN**_**7**_**O**_**2**_ complex. The close agreement between observed and calculated elemental compositions from EDX, coupled with the distinct complex morphologies from SEM, provides strong evidence for the successful synthesis of the desired Schiff base metal complexes [49, 50]. These analyses provide valuable insights into the successful formation of the desired Schiff base complexes and their structural characteristics, which can be further correlated with their potential applications in various fields, such as catalysis, materials science, or biological systems.

#### Molar conductivity measurements

Conductivity assessments serve as a means to evaluate the level of ionization in metal complexes. The investigation into the molar conductivity of metal complexes derived from Schiff bases was systematically examined across various solvents, each chosen based on the solubility of the compounds, utilizing a concentration of (1.0 × 10^−3^ M), conducted at a standardized temperature of 25 °C. The findings distinctly demonstrated that the Cd [II] complexes exhibited characteristics indicative of non-electrolytic behavior [46]. In contrast, the metal complexes involving Co(II), Mn(II), and Cu(II) exhibited electrolytic characteristics, as delineated in Table 1 [51].

#### Mass spectral studies

The mass spectra of the Schiff base **(L**_**1**_) and its ternary metal complexes exhibited distinct molecular ion peaks, as outlined in Table 2. Examination of the mass spectral data verified the stoichiometric composition of the metal complex as **ML**_**1**_**L**_**2**_. Furthermore, the results obtained from these investigations were in complete concordance with the predicted molecular formulas, which were also substantiated by elemental analysis data. A consistent and noteworthy observation in all complex spectra was the presence of the Schiff base ligand (**L**_**1**_) at 318.78 m/z and the glycine (**L**_**2**_) moiety at about 74 m/z. This finding serves as a robust indicator of the complexation process.

**Table 2:** EI- mass data of Schiff base ligand (**L**_**1**_) metal complexes

Compound	m/z value		Interpretation
	Calculated	Found	
**Schiff base ligand (L**_**1**_**)**	318.38	318.78	[M]^+^
**[Mn (L**_**1**_**)(L**_**2**_**)(H**_**2**_**O)].Cl.H**_**2**_**O**	464.39	465	[M + 1]^+^
**[Co(L**_**1**_**)( L**_**2**_**)(H**_**2**_**O)].Cl.H**_**2**_**O**	468.38	468	[M]^+^
**[Cu(L**_**1**_**) (L**_**2**_**)(H**_**2**_**O)].Cl .2H**_**2**_**O**	473	472.8	[M]^+^
**[Cd (L**_**1**_**) (L**_**2**_**)Cl]**	539.30	540	[M + 1]^+^

#### UV–visible absorption spectra for the Schiff Base Ligand (L_1_) and its metal complexes

The electronic spectra of the tridentate Schiff base ligand (**L**_**1**_) and its metal complexes were examined within the 200–700 nm spectral range in a suitable solvent. The UV–Vis spectrum of the standalone Schiff base ligand (**L**_**1**_) displayed three distinct absorption bands at 314, 334, and 388 nm. These absorptions were attributed to different transitions: the first at 314 nm was linked to π-π* transitions, the second at 334 nm signified n-π* transitions within the -C = N- group, while the higher-energy band at 388 nm was associated with charge transfer [52, 53]. Upon complexation with diverse metal cations, the bands observed at 314 nm in the Schiff base ligand (**L**_**1**_) spectrum exhibited shifts to higher or lower wavelengths within the range of 292–316 nm, denoting alterations in the π-π* transitions in all complexes. Furthermore, the band attributed to the azomethine group, observed initially at 334 nm in the ligand spectrum, experienced a shift to the range of 347–369 nm, indicative of n-π* transitions [54]. This spectral data strongly suggests a pronounced implication of the nitrogen azomethine (C = N-) in coordination interaction with the metal ions.

#### ^1^H‐NMR spectral studies

The suggested bonding pattern in the synthesized complexes finds support through ^1^H-NMR spectral analysis. The ^1^H-NMR spectra of the prepared Schiff base ligand **(L**_**1**_**)** and its Cd (II) complex were recorded in DMSO-*d6*, and their respective chemical shift values (ppm) were measured. Multiplet signals at 7.45–8.22 ppm within the ligand spectrum were observed, corresponding to the aromatic group. In comparison, the signal at 8.04 ppm was attributed to the proton of the pyridine ring, as delineated in Table 3. These signals were also evident in the Cd (II) complex, appearing at 7.45–8.49 ppm and 8.36 ppm, respectively. Furthermore, the proton signals from the NH group appeared as singlets at 11.13 and 9.68 ppm in the Schiff base ligand **(L**_**1**_**)**. These signals persisted in the Cd (II) complex but experienced a shift to higher values, registering at 11.27 and 10.031 ppm, respectively. The proton signals from the N–CH = N in pyridine also presented as singlets at 8.49 ppm in the Schiff base ligand **(L**_**1**_**)**. In the Cd(II) complex, these signals remained detectable but shifted to a lower value at 8.36 ppm, indicating the chelation effect (see Table 3). The protons from the methylene and amino groups of the amino acid glycine were observed at 3.54 and 1.35 ppm, respectively. This observation suggests chelation occurred in the Cd(II) complex through Cd binding to the glycine amino group. No signal appeared corresponding to the carboxylic proton of the glycine amino acid, which proves that the depression of the carboxylic proton accompanies the chelation of the glycine amino acid to the metal ions [55, 56].

**Table 3:** presents the ^1^H NMR spectral data for the Schiff base ligand (**L**_**1**_) and its ternary Cd(II) complex

Compound	Chemical shift, (δ) ppm	Assignment
Schiff base ligand (L_1_)	11.13	(s, H, ph-NH)
	9.68	(s, H, NH-Pyridine)
	8.49	(s, H, N–CH = N)
	8.04	(s, H, pyridine)
	7.45–8.22	(m, 8H, aromatic)
	2.07	(s, 3H, N = C-CH_3_)
	2.32	(s, 3H, ph-CH_3_)
**[Cd(L**_**1**_**)(L**_**2**_**)Cl]**	11.27	(s, H, ph-NH)
	10.03	(s, H, NH-Pyridine)
	8.36	(s, H, N–CH = N)
	7.90	(s, H, N = CH)
	7.45–8.49	(m, 8H, aromatic)
	3.54	(s, 2H, CH_2_-C = O)glycine
	2.07	(s, 3H, N = C-CH_3_)
	2.32	(s, 3H, ph-CH_3_)
	1.35	(s, 2H, NH_2_)glycine

#### ^13^C NMR

A direct comparison of the ^13^C NMR spectra of free **L**_**1**_ and its cadmium complex would reveal differences in peak positions and intensities, indicating changes in carbon chemical environments upon metal coordination. This spectral change supports the conclusion that **L**_**1**_ is bound to cadmium in the complex. While simulation of the actual spectra would require unambiguous structural information, the general trends discussed here could be observed experimentally to confirm complex formation. The formation of the cadmium complex with the Schiff base ligand (**L**_**1**_) involves coordination and chelation, evidenced by the shifts observed in the ^13^C NMR spectra. In the cadmium complex **[Cd(L**_**1**_**)(L**_**2**_**)Cl]**, the azomethine carbon (-CH = N-) shifts from 140.0 ppm in the free ligand to 138.8 ppm (1.2 ppm shift) in the complex, suggesting coordination of the azomethine nitrogen to the cadmium center [57]. The appearance of a new peak at 138.8 ppm is attributed to the methylene carbon (-CH_2_-) bridging the Schiff base and the cadmium center, which is absent in the free ligand spectrum. The peak at 169.9 ppm is assigned to the carbon atom of the deprotonated carboxylate group (-COO-) coordinated to the cadmium center [58], not present in the free ligand, indicating deprotonation and coordination. The carbon of the carbonyl group (-C = O) in the glycinate exhibits a significant downfield shift from 178.8 ppm in the free ligand to 169.9 ppm (8.9 ppm shift) in the complex, suggesting the carbonyl oxygen's involvement in cadmium coordination [58]. No shift is observed for methyl, phenyl, and pyridine carbons. The new peaks and shifts provide evidence for chelation of **L**_**1**_ to cadmium, forming a stable five-membered chelate ring involving the azomethine nitrogen and deprotonated glycinate carboxylate.

#### Thermal analysis of ternary Schiff base (L_1_) metal complexes

The study utilized Thermal Gravimetric Analysis (TGA) to assess the synthesized metal complexes' thermal resilience and distinguish between water molecules existing as hydrated or coordinated within their structures [59–64]. Analysis through TG and DTG was conducted on the Schiff base ligand metal complexes, examining their behavior across a temperature spectrum from ambient conditions to 1000 °C. The outcomes encompassed the temperature ranges, decomposition stages, product loss during decomposition, calculated versus observed weight loss percentages, and residues of all compounds, as detailed in Table 4. The thermal analysis of the **[Mn(L**_**1**_**)(L**_**2**_**)(H**_**2**_**O)].Cl.H**_**2**_**O** complex exhibited four decomposition steps. The initial step, between 35 and 105 °C with a peak at 85 °C, led to the loss of hydrated water molecules, estimating a mass loss of 3.05% (calculated = 3.46%). The subsequent step, between 105 and 295 °C with a peak at 245 °C, involved the loss of **C**_**7**_**H**_**10**_**NOCl** with an estimated mass loss of 29.99% (calculated = 30.76%). The third step, between 295 and 445 °C with a peak at 310 °C, indicated the loss of** C**_**2**_**H**_**4**_**NO** with an estimated mass loss of 10.49% (calculated = 11.19%). The final step, between 445 and 800 °C with a peak at 560 °C, related to the loss of **C**_**11**_**H**_**10**_**N**_**5**_ with an estimated mass loss of 40.53% (calculated = 40.90%). After complete decomposition, magnesium oxide **(MnO)** remained as residues. For the **[Co(L**_**1**_**)(L**_**2**_**)(H**_**2**_**O)].Cl.H**_**2**_**O** complex, the first decomposition step occurred between 43 and 103 °C, with a peak at 74 °C, indicating the loss of hydrated water molecules, estimating a mass loss of 3.61% (calculated = 3.70%). The second step occurred from 105 to 505 °C with a peak at 315 °C, involving the loss of **C**_**10**_**H**_**17**_**N**_**2**_**O**_**2**_**Cl** with an estimated mass loss of 43.94% (calculated = 44.55%). The final step, from 505 to 800 °C with a peak at 565 °C, showed the loss of **C**_**10**_**H**_**7**_**N**_**5**_ with an estimated mass loss of 37.31% (calculated = 37.71%), leaving behind cobalt oxide **(CoO)** as the decomposition product. Regarding the **[Cu(L**_**1**_**)(L**_**2**_**)(H**_**2**_**O)].Cl.2H**_**2**_**O** complex, the decomposition occurred in three steps. The first step, between 35 and 125 °C with a peak at 68 °C, accounted for a mass loss of 6.007% (calculated = 6.59%), attributed to the loss of two water molecules. The subsequent step, between 125 and 345 °C with a peak at 225 °C, involved the loss of **C**_**9**_**H**_**14**_**ClN**_**2**_**O**_**2**_ with an estimated mass loss of 39.398% (calculated = 39.70%). The final step, between 345 and 800 °C with a peak at 505 °C, related to the loss of **C**_**11**_**H**_**10**_**N**_**5**_ with an estimated mass loss of 38.16% (calculated = 38.90%). After complete decomposition, copper oxide **(CuO)** remained as residues. In the case of the **[Cd(L**_**1**_**)(L**_**2**_**)Cl]** complex, the first decomposition occurred between 45 and 355 °C, peaking at 275 °C, resulting in the loss of **C**_**9**_**H**_**9**_**ClN**_**3**_ with an estimated mass loss of 35.51% (calculated = 36.02%). The second step occurred between 355–800 °C with a peak at 615 °C, indicating the loss of **C**_**11**_**H**_**13**_**N**_**4**_**O** with an estimated mass loss of 40.11% (calculated = 40.20%), and leaving behind Cadmium oxide **(CdO)** as the product of decomposition.

**Table 4:** Thermal Analysis Findings for Schiff Base Ligand (**L**_**1**_) and Its Metal Complexes via TG and DTG

Complex	TG-range (^o^C)	DTG max	n*	Mass loss Estim (calcd)%	Assignment	Residues
**[Mn(L**_**1**_**)(L**_**2**_**)(H**_**2**_**O)].Cl.H**_**2**_**O** **(C**_**20**_**H**_**26**_**MnClN**_**7**_**O**_**4**_**)**	(35–105)	85	1	3.05 (3.46)	-Loss of H_2_O	MnO
	(105–295)	244	2	29.99 (30.76)	-Loss of C_7_H_10_ClNO	
	(295–446)	310	3	10.49 (11.19)	-Loss of C_2_H_4_NO	
	(445–800)	560	4	40.53 (40.90)	-Loss of C_11_H_10_N_5_	
**[Co(L**_**1**_**)(L**_**2**_**)(H**_**2**_**O)].Cl.H**_**2**_**O** **(C**_**20**_** H**_**26**_**CoClN**_**7**_**O**_**4**_**)**	(45–105)	75	1	3.61 (3.70)	-Loss of H_2_O	CoO
	(105–505)	315	2	43.94 (44.55)	-Loss of C_10_H_17_Cl N_2_O_2_	
	(505–800)	565	3	37.31 (37.71)	-Loss of C_10_H_7_N_5_	
**[Cu(L**_**1**_**) (L**_**2**_**)(H**_**2**_**O)].Cl.2H**_**2**_**O** **(C**_**20**_**H**_**28**_**CuClN**_**7**_**O**_**5**_**)**	(35–125)	70	1	6.007 (6.59)	-Loss of 2H_2_O	CuO
	(125–345)	225	2	39.398 (39.70)	-Loss of C_9_H_14_ClN_2_O_2_	
	(345–800)	505	3	38.16 (38.90)	-Loss of C_11_H_10_N_5_	
**[Cd(L**_**1**_**) (L**_**2**_**) Cl]** **(C**_**20**_**H**_**22**_**CdClN**_**7**_**O**_**2**_**)**	(45–355)	275	1	35.51 (36.02)	-Loss of C_9_H_9_ClN_3_	CdO
	(355–800)	615	2	40.11 (40.20)	-Loss of C_11_H_13_N_4_O	

#### IR spectra

**Fig. 4:**
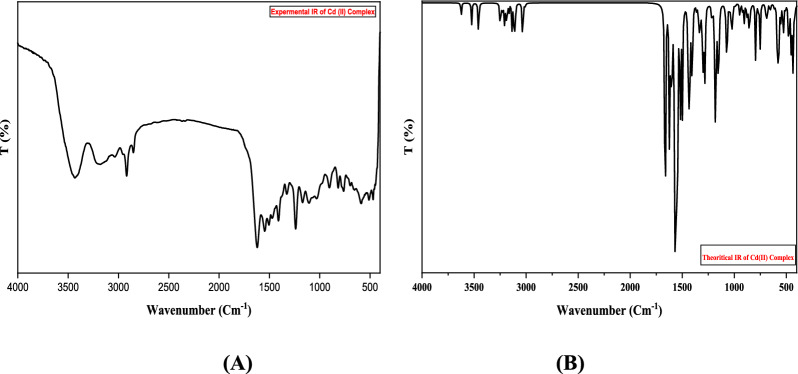
Comparison of **a** Experimental and **b**Theoretical IR Spectra for **[Cd(L**_**1**_**)(L**_**2**_**)Cl]**

To estimate the chelation mode of different ligands to metal (II) ions, the IR spectra of the free primary Schiff base ligand (**L**_**1**_) and secondary ligand glycine (**L**_**2**_) were compared with the IR spectra of the resulting metal complexes [65]. The IR spectrum of the Schiff base ligand displayed three critical characteristic peaks, with the most significant peak appearing at 1605 and 1545 cm^−1^, corresponding to two (C = N) azomethine and (C = N) pyridine, respectively. In the metal complexes, these bands shifted to higher wavenumbers [66]: 1610, 1560 cm^−1^ for Mn(II), 1619, 1545 cm^−1^ for Co (II),1628, 1559 cm^−1^ for Cu(II) and 1620, 1573 cm^−1^ for Cd (II), This shift strongly suggests the involvement of (C = N) groups in coordination [67]. Two other characteristic bands observed in the prepared complex spectra at 1421, 1336 cm^−1^ for Mn(II), 1410, 1324 cm^−1^ for Co(II),1413, 1327 cm^−1^ for Cu(II) and 1422, 1336 cm^−1^ for Cd(II) were associated with (COO^−^) asy and (COO^−^) sym, indicating the participation of the carboxylic group of the glycine ligand in coordination with the metal ions [68, 69]. Additionally, two new distinct bands emerged in all metal complex spectra within the range of 547–560 cm^−1^ and 436–458 cm^−1^, representing the formation of M‐O and M‐N bonds, respectively (Table 5) [70]. The spectra of the Cd (II) complex exhibit distinctive bands at 1550 and 1590 cm^−1^, corresponding to the (C = N) azomethine and (C = N) pyridine moieties, respectively. Also, 1460 and 1340 cm^−1^ peaks indicate (COO −) asymmetric and (COO −) symmetric vibrations, respectively. Furthermore, characteristic signals at 550 cm^−1^ for M–O and 450 cm^−1^ for M–N provide insights into the metal–ligand interactions. Additionally, a broad vibration band attributed to ν (NH) was detected at the range 3430–3437 cm^−1^ due to the coordination of the water molecule OH band and NH stretching vibrations [46]. These spectral features are illustrative examples in theoretical studies, specifically focusing on utilizing Density Functional Theory (DFT), as depicted in Fig. 4. These recorded frequencies align with the theoretical predictions, affirming the successful synthesis of mixed ligand complexes involving a Schiff base ligand (**L**_**1**_). This unity between experimental and theoretical frequencies lends support to the proposed chemical structure of the mixed ligand complex. Discrepancies observed between theoretical and experimental frequencies may be attributed to systematic errors, potentially arising from harmonicity or the utilization of gas-phase molecules in DFT calculations. To address these discrepancies, a correlation coefficient of 0.9648 was applied for LAN2DZ, offering a corrective measure to enhance the accuracy of the theoretical predictions and mitigate the influence of systematic errors [33, 47]. These observations suggest that the prepared Schiff base ligand (**L**_**1**_) acted as a neutral tridentate ligand, coordinating with metal ions via two N‐azomethine and (C = N) pyridine, while the metal coordinated with the glycine moiety through NH_2_ and COO^−^ groups, forming a uninegative bidentate ligand.

**Table 5:** The most significant infrared (IR) spectral bands of the isolated Schiff base ligand (**L**_**1**_) ternary Complexes are as follows

Compound	υ(C = N)	υ(C = N) pyridine ring	υ(COO)_Asymmetric_ glycine	υ(COO)_symmetric_ glycine	υ(M–O)	υ(M–N)
Schiff base ligand (L_1_)	1605 sh	1545 sh	–	–	–	–
[Mn(L_1_)(L_2_)(H_2_O)].Cl.H_2_O	1610 m	1560 sh	1421 sh	1336 sh	506 m	460 w
[Co(L_1_)( L_2_)(H_2_O)].Cl.H_2_O	1619 m	1563 m	1410 m	1324 m	547 m	458 w
[Cu(L_1_)(L_2_)(H_2_O)].Cl.2H_2_O	1628 sh	1559 m	1413 sh	1327 m	560 m	436 w
[Cd(L_1_)(L_2_)Cl]	1620 sh	1573 s	1422 m	1336 sh	586 m	469 m

### Geometry optimization

**Fig. 5:**
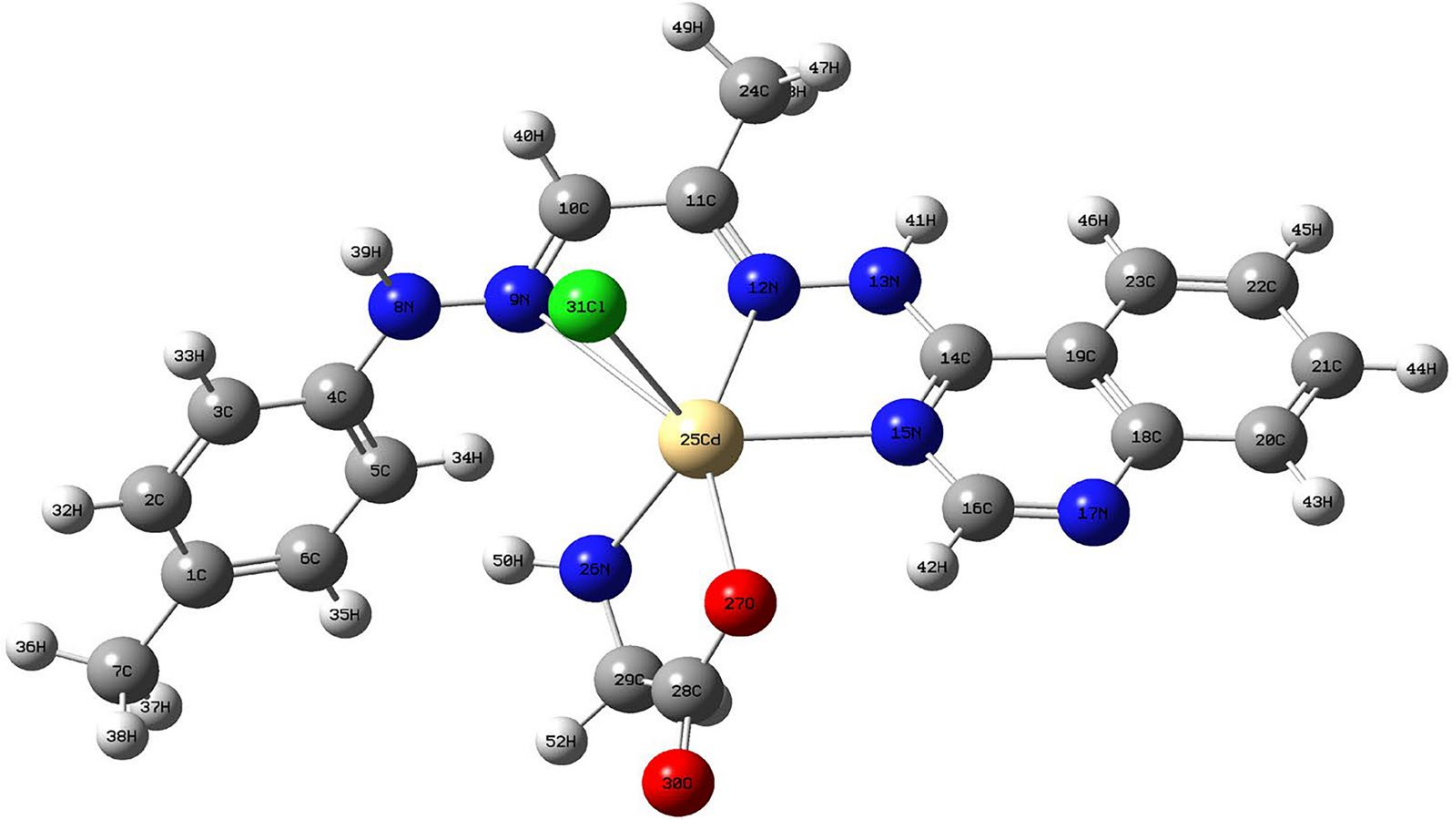
The optimized structure of **[Cd(L**_**1**_**)(L**_**2**_**)Cl]** complex

The geometry optimization was done at the DFT level of theory, and the fully optimized geometry with atomic numbering of the Cd (II) complex was depicted in Fig. 5 [71]. The determined bond lengths and bond angles for the Cadmium [II] complex indicate a distorted octahedral arrangement surrounding the Cd (II) ion, as outlined in Table 6 [72]. The molecular dimensions were computationally determined, precisely the interatomic distances and bond angles of the Schiff base ligand (**L**_**1**_) and its Cadmium(II) complex. A slight elongation in bond lengths N(8)-N(9), N(12)-N(13), and N(15)-C(16) was noted in the Cd(II) complex as a ligand (**L**_**1**_)coordinated via two azomethine nitrogen and azomethine group of pyridine. The main orbital that take place in chemical stability are HOMO (highest occupied molecular orbital) and LUMO (lowest unoccupied molecular orbital). The HOMO represents the electron donor, while LUMO is the electron acceptor. Various supplementary parameters, including the HOMO–LUMO energy gap (ΔE), absolute electronegativities (χ), absolute hardness (η), absolute softness (σ), chemical potentials (Pi), global softness (S), global electrophilicity (ω), and additional electronic charge (ΔNmax), were computed for both the Schiff base ligand (**L**_**1**_) and its Cd (II) complex. The parameters ω + , ω−, and ω are related to the concept of electrophilicity index (ω), which provides a quantitative measure of the electrophilic or nucleophilic behavior of a compound [73, 74]. Where ω + is the electrophilicity index associated with the LUMO acting as an electron acceptor, ω- is the electrophilicity index related to the HOMO acting as an electron donor, and ω is the net electrophilicity index considering both ω + and ω−. The importance of these parameters lies in their ability to predict the reactive behavior of a molecule towards nucleophilic or electrophilic reagents based on the relative stabilities of the compound's LUMO and HOMO levels. ω +  = μ2/2η, where μ is the chemical potential and η is the chemical hardness. A higher ω + value indicates a better ability of the LUMO to accept electrons, making the molecule more electrophilic. ω− = μ2/2(IP-μ), where IP is the ionization potential. A higher ω- value suggests the HOMO can more easily donate electrons, making the molecule more nucleophilic. The overall ω (ω = ω +  + ω−) combines the electrophilic (LUMO) and nucleophilic (HOMO) characteristics into a single descriptor of total electrophilicity. In the context of this study, calculating and analyzing ω + , ω−, and ω can provide valuable insights:

1. Compare the electrophilicities of the ligand vs metal complexes to understand how complexation modulates reactivity.
2. Identify potential electrophilic/nucleophilic reaction sites based on high ω + /ω− values on specific atoms/regions.
3. Correlate electrophilicity differences with observed reactivity trends like biomolecular interactions.

**Table 6:** Optimization and Quantum Chemical Characteristics of Schiff Base Ligand (**L**_**1**_) and its Cadmium (II) Complex

The calculated quantum chemical parameters	Schiff base ligand (L_1_)	**[Cd(L**_**1**_**)(L**_**2**_**)Cl]** complex
E (a.u)	−1018.80	−1371.310
Dipole moment (Debye)	3.9111	15.1873
E_HOMO_ (eV)	−7.4355	−5.863786
E_LUMO_ (eV)	1.4609	−2.766583
ΔE (eV)	8.8964	3.097203
χ(eV)	2.9873	4.3151845
η(eV)	4.4482	1.5486015
σ(eV)^−1^	0.22481	0.645743918
Pi (eV)	−2.9873	−4.3151845
S (eV)^−1^	0.112405	0.322871959
ω(eV)	1.003098	6.01213975
ΔN_max_	0.671575	2.786504146

Bond lengths (Å)	Schiff base ligand (L_1_)	[Cd(L_1_)(L_2_)Cl] complex
N(8)-N(9)	1.337	1.353
N(12)-N(13)	1.351	1.394
N(15)-C(16)	1.344	1.387
N(9)-Cd(25)		3.386
N(12)-Cd(25)		2.531
N(15)-Cd(25)		2.372
Cd(25)-Cl(31)		2.510

Bond angles (o)	Schiff base ligand (L_1_)	[(L_1_)(L_2_)Cd(Cl)] complex
Cl(31)-Cd(25)-O(27)	–	115.32
Cl(31)-Cd(25)-N(26)	–	116.939
Cl(31)-Cd(25)-N(15)	–	124.104
Cl(31)-Cd(25)-N(12)	–	90.783
Cl(31)-Cd(25)-N(9)	–	72.177
O(27)-Cd(25)-N(26)	–	73.444
O(27)-Cd(25)-N(15)	–	83.746
O(27)-Cd(25)-N(12)	–	149.146
O(27)-Cd(25)-N(9)	–	146.129
N(26)-Cd(25)-N(15)	–	118.863
N(26)-Cd(25)-N(12)	–	110.445
N(26)-Cd(25)-N(9)	–	74.062
N(15)-Cd(25)-N(12)	–	67.386
N(15)-Cd(25)-N(9)	–	120.784
N(12)-Cd(25)-N(9)	–	55.01

Guide further molecular design by tuning ω to achieve desired nucleophilic/electrophilic characteristics.

So, in summary, these electrophilicity indices serve as valuable quantum chemical reactivity descriptors that can complement the other calculated parameters in rationalizing structure–activity relationships and directing future compound optimization. The calculations employed specific Eqs. (1–9) outlined in the references [24, 25, 75, 76], and the obtained results have been systematically presented in Table 6:

1 $${\mathbf{I}} \, = \, - \, {\mathbf{E}}_{{{\mathbf{HOMO}}}}$$

2 $${\mathbf{A}} \, = - \, {\mathbf{E}}_{{{\mathbf{LUMO}}}}$$

$${\mathbf{\Delta E}} \, = \, {\mathbf{E}}_{{{\mathbf{LUMO}}}} - \, {\mathbf{E}}_{{{\mathbf{HOMO}}}}$$

3 $${{\varvec{\upchi}}}\, = \, - \,\frac{{E_{LUMO} \, + \,E_{HOMO} }}{2}\, = \,\frac{I\, + \,A}{2}$$

4 $$\varvec{\eta} \, = \,\frac{{E_{LUMO} \, - \,E_{HOMO} }}{2}\, = \,\frac{I\, - \,A}{2}$$

5 $$w\, = \,\frac{{\left( {I\, + \,A} \right)^{2} }}{{4\,\left( {I\, - \,A} \right)}}$$

6 $$w^{ - } \, = \,\frac{{\left( {3I\, + \,A} \right)^{2} }}{{16\,\left( {I\, - \,A} \right)}}$$

7 $$w^{ + } \, = \,\frac{{\left( {I\, + \,3A} \right)^{2} }}{{16\,\left( {I\, - \,A} \right)}}$$

8 $${{\varvec{\upsigma}}} \, = \, {\mathbf{1}}/{{\varvec{\upeta}}}$$

9 $${\mathbf{\Delta N}}_{{{\mathbf{max}}}} \, = \, - {\mathbf{I}}/{{\varvec{\upeta}}}$$

The gap energy (ΔE) is a crucial metric for assessing stability, elucidating the structural intricacies and conformational hindrances within numerous molecular systems. This energy gap was 8.89 eV for the Schiff base ligand (**L**_**1**_) and 3.097 eV for the Cd (II) complex. When this energy increases, the compound becomes more stable. Moreover, the chemical potential (Pi) exhibited a negative value, whereas the electrophilicity index (χ) demonstrated a positive numerical representation [22, 23]. These indicate that the Schiff base ligand might donate electrons to metal ions [77]. The higher HOMO energy of the complex shows that it is easier to remove an electron from the complex compared to the ligand, suggesting increased reactivity. The lower LUMO energy of the complex indicates that adding an electron to the complex is more accessible than the ligand, suggesting increased reactivity. The smaller energy gap of the complex indicates a higher degree of reactivity and potential for charge transfer or electronic transitions than the ligand [78, 79].

### Electron distribution characterization via Molecular Electrostatic Potential (MEP)

**Fig. 6:**
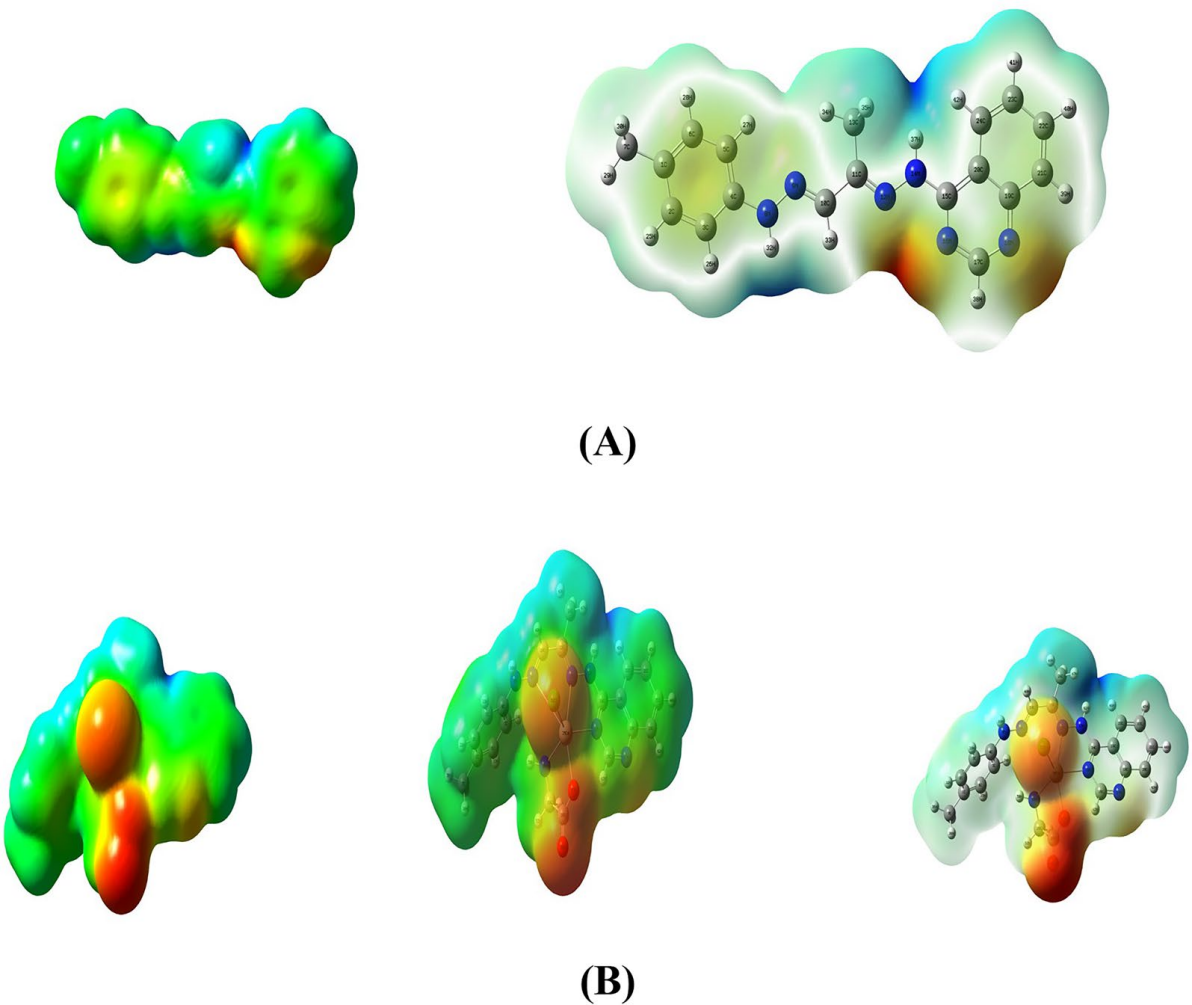
Molecular electrostatic mapping potential of **A** Schiff Base Ligand (**L**_**1**_) and **B [Cd(L**_**1**_**)(L**_**2**_**)Cl]** Complex. The Isosurface Represents an electron density of 0.004 Atomic Units

Electrostatic potential V(r) mappings were computed to explore the dynamic responses of molecular systems. These mappings serve as valuable tools for discerning the electronic charge distribution encompassing the molecular surface, facilitating the anticipation of reaction sites [80]. The cartography of molecular electrostatic potential (MEP) for both the Schiff base ligand (**L**_**1**_) and its Cd(II) complex was derived using the identical basis set employed during the optimization process. The generated three-dimensional MEP plots are depicted in Fig. 6. Analysis of the MEP allows for delineating electron-rich regions, denoted by a red coloration on the map, indicative of favorable sites for electrophilic attacks. Conversely, blue regions represent electron-poor areas, suggesting susceptibility to nucleophilic attacks [70]. The area characterized by green-colored markers signifies a region of electrostatic potential neutrality. It can be seen that the Schiff base ligand **(L**_**1**_**)** is stable, having an almost uniform distribution of charge. These electrostatic potential V(r) maps showed that chelation enhances the reaction site in **L**_**1**_ metal complexes. In the ligand structure, we can observe that the oxygen atoms and the imine nitrogen atoms exhibit a higher negative electrostatic potential (red regions), indicating that these atoms are likely to be the nucleophilic sites or electron-rich centers within the ligand. The aromatic rings and alkyl groups show a more neutral or slightly positive electrostatic potential (blue regions). In the molecular electrostatic potential map of the **[Cd(L**_**1**_**)(L**_**2**_**)Cl]** complex, we can observe a more pronounced polarization of the electrostatic potential compared to the free ligand. The chloride ion (Cl^−^) exhibits a highly negative electrostatic potential (intense red region), indicating its strong electronegativity and high electron density around this ion. This negative charge is balanced by the positive charge on the cadmium ion (Cd^2+^), represented by a blue region, indicating a lower electron density or positive electrostatic potential. The ligands (**L**_**1**_ and **L**_**2**_) coordinated with the cadmium ion also show distinct regions of negative electrostatic potential around the oxygen and imine nitrogen atoms, similar to the free ligand (**L**_**1**_). However, these negative regions appear more polarized or enhanced due to the cadmium ion's influence and the complex's overall charge distribution [81].

### Antimicrobial activities

**Fig. 7:**
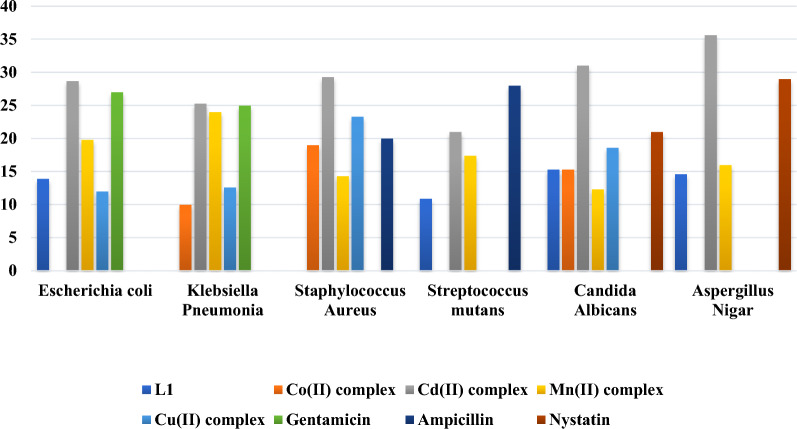
Antimicrobial activity of Schiff base ligand (**L**_**1**_) and its associated ternary complexes

Compared to the free Schiff base ligands, the enhanced antibacterial efficacy observed in metal complexes can be attributed to the chelation process, where the Schiff base coordinates with metal ions, endowing the resulting metal chelates with polar and nonpolar properties [82]. This property is advantageous as it facilitates the penetration of these complexes into cells and tissues. The diminished metal ion polarity results notably from the overlap of ligand orbitals during chelation, enabling a partial distribution of the positive charge of the metal ion among the coordinating donor groups. Consequently, chelation induces heightened π-electron delocalization across the entire chelate ring, thereby enhancing the permeability of the complexes through lipid membranes [83, 84]. Additionally, chelation processes augment the hydrophilic and lipophilic characteristics of central metal ions, potentially amplifying their lipid-soluble properties and facilitating their penetration through the lipid bilayer of cellular membranes. The coordination also alters lipophilicity, influencing the rate at which molecules enter cells. Consequently, the modified nature of the metal complex, as a result of coordination, renders it more active than the free Schiff base ligand [83, 85]. The compounds prepared were tested for their antimicrobial properties against various strains of bacteria and fungi. The assessment involved Gram (+ ve) bacteria, namely *Streptococcus mutans* and *Staphylococcus aureus*, Gram (−ve) bacteria, including *Escherichia coli* and *Klebsiella pneumonia*, as well as two strains of fungi *Aspergillus niger* and *Candida albicans*. The detailed outcomes of these evaluations were tabulated in Table 7 and graphically depicted in Fig. 7. The collected data revealed that the Schiff base ligand **(L**_**1**_**)** exhibited biological efficacy against various bacterial species, except for *Klebsiella pneumonia* and *Staphylococcus aureus*. The study further investigated the in-vitro antibacterial and antifungal activities of the Schiff base ligand and its metal complexes. Standard antiseptic, antibiotic, and antifungal drugs such as ampicillin, gentamycin, and nystatin were employed for comparison. Upon analyzing the biological effects of the Schiff base ligand **(L**_**1**_**)**, metal (II) complexes, and standard drugs, a notable observation emerges: all the complexes exhibit superior inhibitory effects on bacterial growth compared to the original ligand. Several insights can be drawn from the antibacterial test results (Table 7, Fig. 7):

1. The complexes' observed antibacterial efficacy is attributed to imine moieties (-C = N) within them. It is suggested that their mechanism of action involves the creation of hydrogen bonds via the (C = O) azomethine group, which interacts with vital cellular components. This interaction may disrupt normal cellular processes, contributing to the compounds' antibacterial properties.
2. Chelation with M(II) ions results in decreased polarity as the positive charge of the metal ion is neutralized by the ligand-donor groups. This process enhances the hydrophobic and lipophilic nature of the ligands, facilitating their permeation through the lipid layers of cell membranes. This penetration can lead to the deactivation of enzymes involved in cellular respiration and the inhibition of protein synthesis, effectively restricting the growth of the organisms.
3. The data suggests that the Cd-complex exhibits higher toxicity against bacterial species than most antibiotics, except for cases where its potency is less than that of ampicillin against *Streptococcus mutans*.
4. In terms of antifungal activity against *Candida albicans,* the compounds' effectiveness ranks in the following order:

**Table 7:** Bioactivity Evaluation of Schiff Base Ligand (**L**_**1**_) and Its Corresponding Metal(II) Complexes

Sample Microorganism	L_1_	(1) [Mn(L1)(L2)H2O].Cl.H2O	(2) [Co(L_1_)(L_2_)H_2_O].Cl.H_2_O	(3) [Cu(L_1_)(L_2_)H_2_O].Cl.2H_2_O	(4) [Cd(L_1_) (L_2_)Cl]	Standard drug
Gram-negative bacterial						Gentamicin
*Escherichia coli* (ATCC: 10536)	13.9 ± 1.0	19.8 ± 0.5	NA	12.0 ± 1.0	28.7 ± 0.5	27 ± 0.5
*Klebsiella Pneumonia* (ATCC: 10031)	NA	24.0 ± 1.0	10 ± 0.5	12.6 ± 0.5	25.3 ± 0.5	25 ± 0.5
Gram-positive bacteria						Ampicillin
*Staphylococcus Aureus* (ATCC: 13565)	NA	14.3 ± 0.5	19 ± 0.6	23.3 ± 0.6	29.3 ± 0.6	20 ± 0.1
*Streptococcus mutans* (ATCC: 25175)	10.9 ± 0.5	17.4 ± 1.0	NA	NA	21.0 ± 1.0	28 ± 0.5
Fungi						Nystatin
*Candida Albicans* (ATCC: 10231)	15.3 ± 0.5	12.3 ± 0.5	15.3 ± 0.5	18.6 ± 0.6	31.0 ± 1.0	21 ± 0.5
*Aspergillus Nigar* (ATCC: 16404)	14.6 ± 0.5	16 ± 0.5	NA	NA	35.6 ± 0.5	29 ± 0.5

**{[Cd(L**_**1**_ **)(L**_**2**_ **)(Cl)]) > Nystatin > ([Cu(L**_**1**_ **)(L**_**2**_ **)(H**_**2**_ **O)].Cl 0.2H**_**2**_ **O) > (L**_**1**_ **) = ( [Co(L**_**1**_ **)(L**_**2**_ **)(H**_**2**_ **O)].Cl.H**_**2**_ **O)) > ([Mn (L**_**1**_ **)(L**_**2**_ **)(H**_**2**_ **O)].Cl.H**_**2**_ **O)}.**

#### Structure–Activity Relationship (SAR)

Some key structure–activity trends can be observed from the antimicrobial data:

1. The cadmium(II) complex was the most potent overall against gram-positive bacteria and fungi, indicating the critical role of the more significant, softer Cd(II) metal center [86].
2. The cobalt(II) complex demonstrated strong and selective activity against gram-negative bacteria like E. coli and K. pneumoniae. The unique properties of the kinetically inert Co(II) oxidation state may be responsible [87].
3. The manganese(II) complex showed limited antimicrobial efficacy, suggesting manganese may not be an optimal metal center for these ligand systems [88, 89].
4. Substituting different metal centers into the same ligand framework resulted in diverse antimicrobial profiles, highlighting how metal geometry, charge, and electronic properties can significantly influence bioactivity [90, 91].

These findings suggest that the complexes, formed by the Schiff base ligand with metal(II) ions, exhibit enhanced antibacterial and antifungal properties compared to their individual components, owing to their unique mechanisms of action and chemical characteristics. Antibacterial action depends on various factors beyond chelation. The effectiveness against microbes is significantly influenced by several crucial elements, including the metal ion's characteristics, ligand properties, coordination sites, complex geometry, concentration, hydrophobicity, and co-ligands' presence. Suppose the geometric and charge distribution around a substance did not match the configuration within the bacterial cell wall pores. In that case, it cannot penetrate the wall, compromising its compatibility and preventing toxic reactions inside the pores. This discrepancy might elucidate the weaker activity of specific complexes [92–97]. Some activities observed could result from the damage incurred during cell wall synthesis, altering the cell's permeability and ultimately leading to cell death [98].

### Bioactive inhibition targeting *H. pylori*

**Fig. 8:**
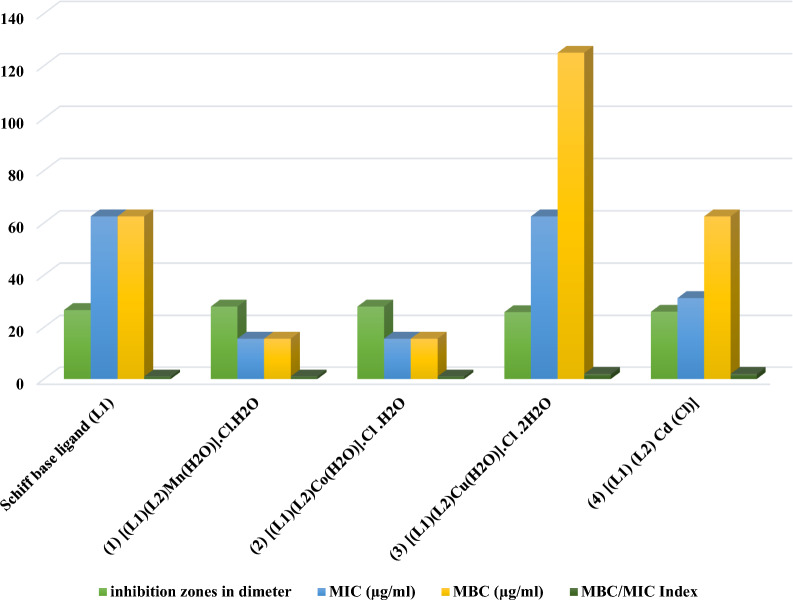
The inhibitory potential of Schiff base ligand (**L**_**1**_) and its metal complexes against *H. pylori*

The study aimed to assess the inhibitory potential of Schiff base ligand **(L**_**1**_**)** and its metal complexes against *H. pylori* by measuring inhibition zones, as indicated in Table 8 [19]. Furthermore, the investigation included the determination and presentation of the Minimum Inhibitory Concentration (MIC) and Minimum Bactericidal Concentration (MBC) for both the Schiff base ligand (**L**_**1**_) and its corresponding metal complexes. The results of these analyses have been provided in Table 8 and visually represented in Fig. 8 (where the MIC test demonstrates the lowest level of antimicrobial agent that significantly inhibits growth, and the MBC demonstrates the lowest level of antimicrobial agent resulting in microbial death). The MBC/MIC index delineated in Table 8 and illustrated in Fig. 8 was employed to classify the samples' bactericidal or bacteriostatic efficacy. If the MBC/MIC index was ≤ 4, it indicated bactericidal activity, while an index > 4 suggested bacteriostatic activity [42]. Table 8 shows that all synthesized compounds exhibited substantial anti-*H—Pylori* activity. In Table 9, the synthesized compounds were found to have varying degrees of effectiveness in inhibiting H. pylori, with the order of efficacy as follows: **[Co(L**_**1**_**)(L**_**2**_**)(H**_**2**_**O)].Cl.H**_**2**_**O = [Mn(L**_**1**_**)(L**_**2**_**)(H**_**2**_**O)].Cl.H**_**2**_**O > Schiff base ligand (L**_**1**_**) > [Cd (L**_**1**_**)(L**_**2**_**)Cl] > [Cu(L**_**1**_**)(L**_**2**_**)(H**_**2**_**O)]. Cl.2H**_**2**_**O.**

**Table 8:** Minimum inhibition and minimum bacteriostatic/bactericidal concentration of Schiff base ligand (**L**_**1**_) and its associated metal complexes against *H. pylori*

Compounds	MIC (µg/ml)	MBC (µg/ml)	MBC/MIC Index
Schiff base ligand (**L**_**1**_)	62.5	62.5	1
**[Mn(L**_**1**_**)(L**_**2**_**)(H**_**2**_**O)].Cl.H**_**2**_**O**	15.62	15.62	1
**[Co(L**_**1**_**)( L**_**2**_**)(H**_**2**_**O)].Cl.H**_**2**_**O**	15.62	15.62	1
**[Cu(L**_**1**_**)(L**_**2**_**)(H**_**2**_**O)].Cl.2H**_**2**_**O**	62.5	125	2
**[Cd(L**_**1**_**)(L**_**2**_**)Cl]**	31.25	62.5	2

**Table 9:** The diverse inhibitory capacities against *H. pylori* demonstrated by Schiff base ligand (**L**_**1**_) and its associated metal complexes

Compounds	Read1	Read2	Read3	Mean	SD	SE
Schiff base ligand (**L**_**1**_)	27.00	26.50	26.50	26.67	0.29	0.09
**[Mn(L**_**1**_**)(L**_**2**_**)(H**_**2**_**O)].Cl.H**_**2**_**O**	28.00	28.00	28.00	28.00	0.00	0.00
**[Co(L**_**1**_**)(L**_**2**_**)(H**_**2**_**O)].Cl.H**_**2**_**O**	28.00	28.00	28.00	28.00	0.00	0.00
**[Cu(L**_**1**_**)(L**_**2**_**)(H**_**2**_**O)].Cl.2H**_**2**_**O**	26.00	26.00	25.50	25.83	0.29	0.09
**[Cd(L**_**1**_**) (L**_**2**_**)Cl]**	26.00	26.00	26.00	26.00	0.00	0.00
Positive control	24.00	24.00	24.50	24.17	0.29	0.09

The data suggests that certain synthesized compounds, particularly **[Co(L**_**1**_**)(L**_**2**_**)(H**_**2**_**O)].Cl.H**_**2**_**O** and **[Mn(L**_**1**_**)(L**_**2**_**)(H**_**2**_**O)].Cl.H**_**2**_**O** displayed significant inhibitory effects against *H. pylori*. This inhibition is likely attributed to the interaction of the metal centers (Co and Mn) in these Schiff base complexes with essential biomolecules of *H. pylori*, disrupting its metabolic processes and growth. Consequently, the presence of the Schiff base ligand **(L**_**1**_**)** and its coordination with transition metals like Co (II) and Mn (II) appears to confer inhibitory properties against *H. pylori*. These findings exhibit potential for creating innovative therapeutic agents in chemotherapy, potentially providing alternative or complementary treatments for *H. pylori* infections.

### Anticancer activities

**Fig. 9:**
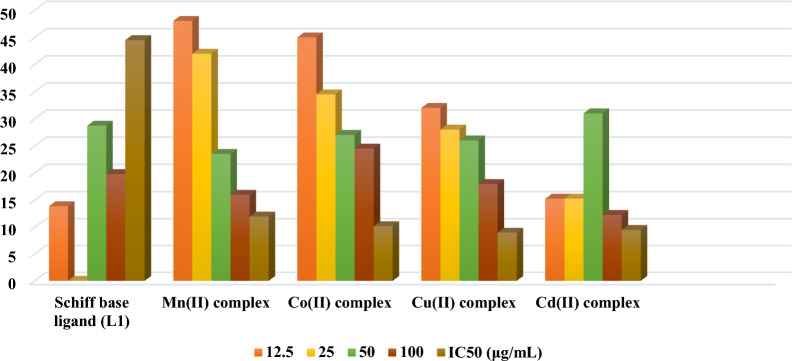
Anticancer activity against breast cancer of the Schiff bases ligand (**L**_**1**_) and its metal complexes

Presently, pharmaceutical research stands at the forefront of discovering novel active drugs for treating cancer. Among the critical areas in this field, the development of metal-based anticancer drugs holds significant importance. Numerous transition metal complexes have been synthesized and analyzed for their potential anticancer properties [99]. In laboratory settings, in vitro studies were carried out to assess the cytotoxicity of a ligand and its complexes against MCF-7 (Breast carcinoma) cells. The relationship between the compounds' concentrations and the cells' relative viability was graphed, producing survival curves for each tumor cell line Fig. 9. Table 10 displays the IC_50_ values, representing the concentration causing a 50% inhibition of cancer cell growth. The observations regarding cytotoxicity against MCF-7 (Breast carcinoma) cells are as follows:

A) Metal complexes of the Schiff base ligand, specifically Mn(II), Co(II), Cu(II), and Cd(II) complexes, demonstrated significant activity against cancer cells, with IC_50_ values of 12, 10.2, 9 and 9.5 μg/mL, respectively. These findings classify these compounds as significant in chemotherapy, where IC50 reflects the concentration causing a 50% inhibition in cancer cell growth.
B) The sequence of potency concerning the chelated metal ions with the ligand follows the order Schiff base Ligand **L**_**1**_ < Mn(II) < Co(III) < Cd(II) < Cu(II) against MCF-7 cells.
C) Comparative analysis of the antitumor activities of the free Schiff base ligand (**L**_**1**_) and its metal complexes suggests that the Cu-complex exhibits superior antitumor activity compared to the ligand and its other metal complexes (Table 10).
D) The Schiff base ligand (**L**_**1**_) displayed minimal cytotoxic effects on the investigated cancer cell line, while the Cu(II) complex showed notably cytotoxic solid effects.
E) A discernible correlation exists between antitumor activities and antimicrobial activities. For instance, the Cd-complex demonstrated elevated antifungal and antibacterial activity against the investigated fungal and bacterial species while exhibiting robust antitumor activities against MCF-7 (Breast carcinoma) cells (refer to Table 10).
F) The complex **[Cu(L**_**1**_**)(L**_**2**_**)(H**_**2**_**O)].Cl.2H**_**2**_**O** was identified as the most active and effective complex, potentially suitable as an anti-neoplastic agent targeting breast carcinoma. The heightened efficacy of the synthesized compounds may be ascribed to the existence of the azomethine group within the macrocyclic chelate ring. The coordination of metal ions with the nitrogen atom of the azomethine group in the chelate ring leads to a reduction in the polarity of the metal atoms. This phenomenon is likely a consequence of the partial sharing of positive charge between the metal ions and the ligand [100–102]. The observed increase in activity can be attributed to the augmented π-electron delocalization within the ligand moiety upon complexation. Consequently, the penetration of the complexes through cell membranes' lipid layers might be augmented, making it more efficient [103].

**Table 10:** Anticancer activity against breast cancer of Schiff base ligand (**L**_**1**_) and its metal complexes

Compounds	Conc. (μg/mL)				IC_50_ (μg/mL)
	12.5	25	50	100	
	Surviving fraction (MCF7)				
Schiff base ligand (**L**_**1**_)	92	72	44	39.5	44.5
**[Mn(L**_**1**_**)(L**_**2**_**)(H**_**2**_**O)].Cl.H**_**2**_**O**	48	42	23.5	16	12
**[Co(L**_**1**_**)( L**_**2**_**)(H**_**2**_**O)].Cl.H**_**2**_**O**	45	34.5	27	24.5	10.2
**[Cu(L**_**1**_**)(L**_**2**_**)(H**_**2**_**O)].Cl.2H**_**2**_**O**	32	28	26	18	9
**[Cd(L**_**1**_**)(L**_**2**_**)Cl]**	36	27	25	19	9.5

The IC_50_ value (µg/mL) signifies the concentration of a drug required to impede the growth of cancer cells by 50%, denoting its cytotoxic dose

The IC_50_ range (µg/mL) categorizes potency levels as follows: 1–10 (very potent), 11–20 (potent), 21–50 (moderately potent), 51–100 (mildly potent), and above 100 (non-cytotoxic) [104]

Specifically referencing MCF-7, a type of carcinoma breast cell line

#### Cytotoxicity and structure–activity relationships

The in vitro cytotoxic activities of the Schiff base ligand (**L**_**1**_) and its corresponding metal complexes were evaluated against the MCF7 breast cancer cell line using the MTT assay. The IC_50_ values, representing the compound concentrations causing 50% cell death, provide insights into their potencies as potential anticancer agents. The free ligand **L**_**1**_ exhibited modest cytotoxicity with an IC_50_ of 44.5 μg/mL. However, upon metal complexation, the bioactivities were significantly enhanced. The manganese(II) complex **[Mn(L**_**1**_**)(L**_**2**_**)(H**_**2**_**O)].Cl.H**_**2**_**O** showed an IC_50_ of 12 μg/mL, a nearly fourfold increase compared to the free ligand. This suggests the Mn(II) metal center is important in augmenting the cytotoxic properties. Even greater potency was observed for complexes 2–4 containing the heavier Co(III), Cu(II), and Cd(II) metal ions, with IC_50_ values of 10.2, 9.0, and 9.5 μg/mL, respectively. The high oxidizing Co(II) state likely contributes to enhanced redox activation and generation of reactive oxygen species. The soft, thiophilic nature of the Cu(II) and Cd(II) centers in **[Cu(L**_**1**_**)(L**_**2**_**)(H**_**2**_**O)].Cl.2H**_**2**_**O** and **[Cd(L**_**1**_**)(L**_**2**_**)Cl]** may facilitate targeting biomolecules like DNA or disrupting redox homeostasis. A notable structure–activity trend is that the cytotoxicities increase progressively from the manganese(II) to cobalt(III) to copper(II)/cadmium(II) complexes. This correlates well with the Irving-Williams order of increasing cellular uptake and biomolecular binding affinities for divalent metal ions: Mn(II) < Co(II) < Cu(II) > Cd(II). The chloride and aqua co-ligands may also influence bioactivity through effects on hydrophobicity, cellular permeability, and rates of ligand substitution reactions. Overall, this study demonstrates that metal coordination can profoundly impact the anticancer properties of the Schiff base scaffolds. The potent cytotoxicities of the Schiff base complexes against MCF7 cells warrant further investigation as potential chemotherapeutic leads. Additional mechanistic studies probing their modes of action, tumor selectivities, and in vivo efficacies are merited to evaluate their therapeutic potential fully.

### Docking study

The potential interaction sites between Schiff base ligand **L**_**1**_ and its **[Cd(L**_**1**_**)(L**_**2**_**)Cl]** complex were examined across seven distinct receptors. These include **(PDB ID: 3AHU)** the crystal structures of the outer membrane protein G of YmaH (Hfq) from *Bacillus subtilis* in complex with an RNA aptamer. Bacterial Hfq is a protein that is vital in regulating genes in cooperation with sRNAs [105]. The crystal structure of the outer membrane protein G of the ACYL-ENZYME complex of *Staphylococcus aureus* BETA-LACTAMASE **(PDB ID: 1GHP).** Beta-lactamases are enzymes produced by some bacteria, including Staphylococcus aureus, that confer resistance to beta-lactam antibiotics (such as *penicillins* and cephalosporins) by hydrolyzing the beta-lactam ring. Understanding the structural details of these enzymes and their interactions with antibiotics is crucial for developing strategies to overcome antibiotic resistance [106]. The crystal structure of *Streptococcus mutans* dextran glucosidase (**PDB ID: 2ZIC**), where Dextran glucosidase is an important enzyme involved in the breakdown of dextran, a glucose polymer produced by *S. mutans* [107]. The crystal structure of this enzyme provides valuable insights into its structure and function, which are important for the following reasons: (A) Dental caries (tooth decay): *Streptococcus mutans* is a major causative agent of dental caries, one of the most common infectious diseases globally. (B) The ability of *S. mutans* to produce and metabolize dextran is crucial for forming dental plaque, a sticky biofilm that adheres to tooth surfaces and contributes to the development of caries. Complex II (Succinate Dehydrogenase) from *E. coli* with ubiquinone bound (**PDB ID: 1NEK**), Where Complex II is an important enzyme complex that plays a crucial role in the citric acid cycle (TCA cycle) and the electron transport chain in cellular respiration [108]. Yeast-specific serine/threonine protein phosphatase (PPZ1) of *Candida albicans* (**PDB ID: 5JEP**), Where PPZ1 is an important enzyme that plays a crucial role in the regulation of various cellular processes in *C. albicans*, a major fungal pathogen responsible for invasive candidiasis and other opportunistic infections in humans. PPZ1 is a potential target for developing new antifungal drugs, as it is essential for the survival and pathogenicity of *C. albicans* [109]. Microtubule (EPB) in complex with tubulin, a crucial component of the cytoskeleton and a significant target in cancer treatment (**PDB ID: 7DAE**) [110]. The structural information provided by this crystal structure is important for the following reasons: (**A**) Microtubule dynamics and function: Microtubules are dynamic polymers that play essential roles in various cellular processes, including cell division, intracellular transport, and maintenance of cell shape. The crystal structure of the microtubule-tubulin complex provides insights into the molecular architecture and interactions that govern microtubule assembly, stability, and dynamics. (**B**) Cancer therapy targeting: Microtubules are a significant target for many anticancer drugs, such as taxanes (e.g., paclitaxel) and vinca alkaloids (e.g., vincristine), which interfere with microtubule dynamics and disrupt cell division. The structural information from **7DAE** can aid in understanding the binding modes and mechanisms of action of these drugs and guide the development of new and improved microtubule-targeted anticancer agents [110]. The structural conformation of the receptor binding domain (RBD) of SARS-CoV-2 is bound intricately with the human antibody CR3022 (**PDB ID: 6W41**) [111]. This structure provides crucial insights into the interaction between the viral protein and the neutralizing antibody, and its importance can be highlighted as follows: (A) Understanding viral entry and neutralization: The RBD of the SARS-CoV-2 spike protein is responsible for binding to the human ACE2 receptor, facilitating viral entry into host cells. The structural information from **6W41** reveals the specific interactions between the RBD and the neutralizing antibody CR3022, shedding light on the mechanisms by which antibodies can block viral entry and neutralize the virus [111]. (B) Antibody-based therapeutics and vaccine design: The structural details of the RBD-antibody complex can guide the design and development of antibody-based therapeutics and vaccines against SARS-CoV-2. Researchers can engineer or optimize antibodies with enhanced neutralizing potency, breadth, and specificity by understanding the molecular basis of antibody neutralization. Molecular docking for these seven distinct receptors interact with Schiff base ligand (**L**_**1**_) and its Cd(II) Complex was carried out by using the MOE 2014.09 program (MOE 2014.09 [112]; The computational software developed by the Chemical Computing Group in Montreal, Canada, is employed for elucidating and forecasting the binding conformation within the protein pockets. The computational operations were executed on a computing system featuring an Intel Pentium processor running at a clock speed of 1.6 GHz, with 512 MB of memory, and equipped with the Windows XP operating system. The minimization procedures employed the Molecular Operating Environment (MOE) until reaching a root mean square deviation (RMSD) gradient of 0.05 kcal/mol Å. The molecular mechanics force field MMFF94X was applied during these optimizations, and the determination of partial charges was automatically computed. In Tables 11 and 12, we present two-dimensional (2D) and three-dimensional (3D) plots illustrating the docking results of Schiff base ligand **L**_**1**_ and its **[Cd(L**_**1**_**)(L**_**2**_**)Cl]** complex, respectively. Additionally, the binding energies for Schiff base ligand **L**_**1**_ and its Cd (II) complex were calculated and are provided in Table 13 for **L**_**1**_ and Table 14 for the **[Cd(L**_**1**_**)(L**_**2**_**)Cl]** complex. Experimental anticancer activities data indicated that Schiff base ligand had IC_50_ value = 44.5 μg/against breast cancer cell line, and its Cu (II) and Cd (II) complex had the lowest IC_50_ values, 9 and 9.5 μg/mL, respectively. To confirm this data, theoretical docking studies were investigated against Microtubule (EPB) in a complex with tubulin, a crucial cytoskeleton component and a significant target in cancer treatment (**PDB ID: 7DAE**). The docking results showed that the lowest binding energies of Schiff base ligand **L**_**1**_ and its Cd (II) complex were—1.1 and −6.8 kcal mol^−1^, respectively. These indicated the more active the complex than its parent ligand, and this large decrease occurred due to the formation of coordination bonds with the Cd (II) ion. Due to the significant activities observed in these compounds were further examined for interactions with various outer membrane proteins from different bacteria, fungi, and the SARS-CoV-2 receptor. The findings revealed substantial and effective interactions between the prepared compounds and these receptors. The primary interaction forces observed between the compounds and the active sites were H‐donor, H‐acceptor, and π‐H. Notably, the lowest binding energies of Schiff base ligand **L**_**1**_ and its Cd (II) complex with **3AHU, 1GHP, 2ZIC, 1NEK, 5jpe,** and **6W41** receptors were recorded as NA, − 2.5, − 1.3, − 0.8, − 5.9, and − 2.1 kcal mol^−1^ for the Schiff base ligand **L**_**1**_, and − 2.4, − 3, − 2.8, − 2, − 10.2,

**Table 11:**
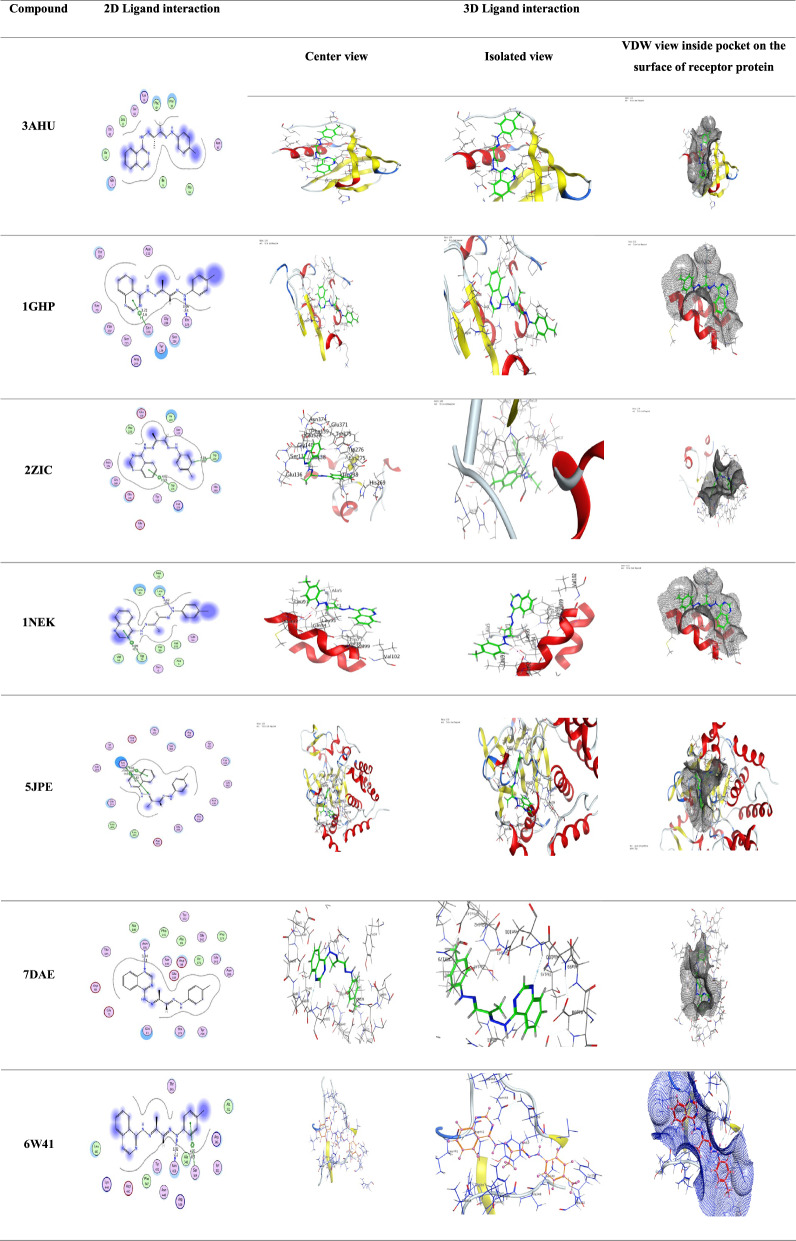
Molecular docking 2D and 3D for predicting the possible binding modes of the studied Schiff base ligand (**L**_**1**_) with the receptors of the crystal structure from the Protein Data Bank of different types of bacterial species and fungi

In addition, PDB co-crystal forms for cancer cell protein

**Table 12:**
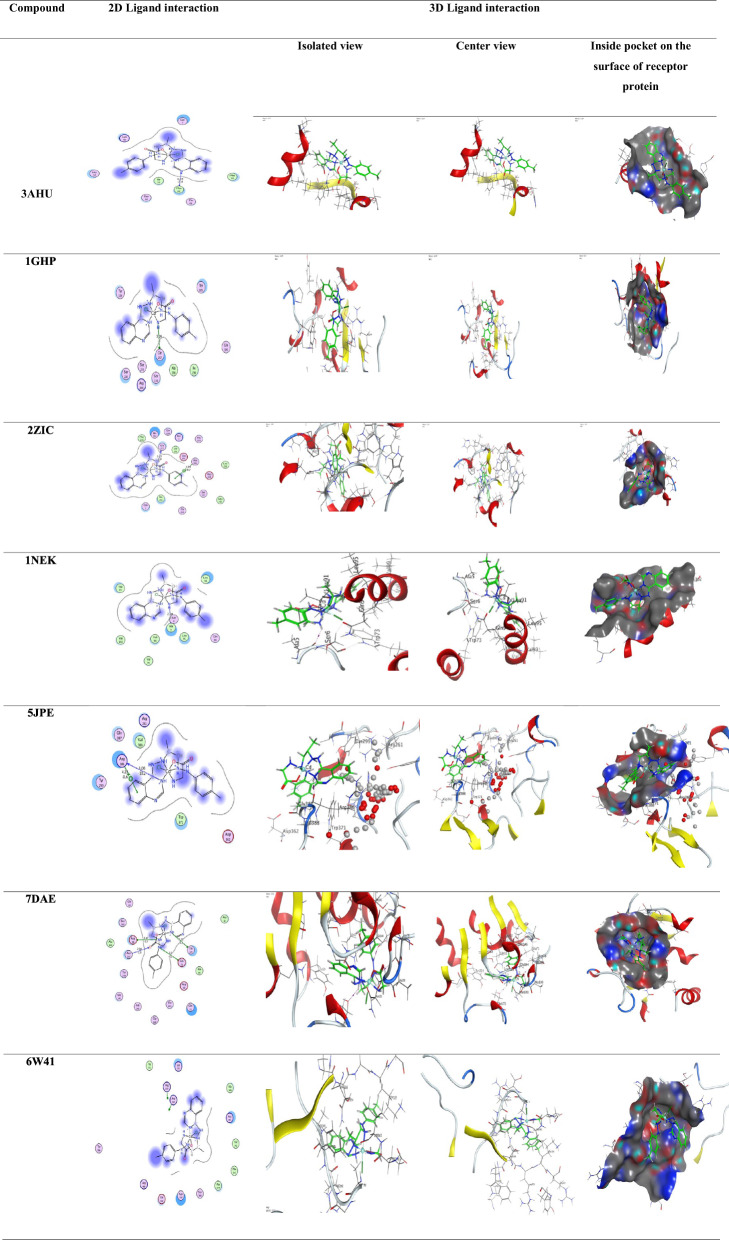
Molecular docking 2D and 3D for exploring the binding configurations of the studied Cadmium [II] complex with the receptors crystal structure from the Protein Data Bank of different types of bacterial species and fungi

In addition, PDB co-crystal forms for cancer cell protein

**Table 13:** Interaction energy values obtained from docking calculations of Schiff base ligand (**L**_**1**_) with different protein receptors

Type	Receptor	Ligand moiety	Receptor site	Interaction	Distance (A^ο^)	E (kcal/mol)
G^+^ Bacteria	3AHU	–	–	–	–	–
	1GHP	N 8	O THR 128 (A)	H-donor	3.06	−2.5
		6-ring	OG SER 130 (A)	pi-H	3.72	−1.6
	2ZIC	C 2	6-ring TRP 238 (A)	H-pi	4.55	−0.6
		6-ring	NE1 TRP 276 (A)	pi-H	4.62	−1.3
G^−^ Bacteria	1NEK	N 8	O LEU 91 (D)	H-donor	3.42	−0.8
		6-ring	CH2 TRP 73 (D)	pi-H	4.09	−0.6
Fungi	5jpe	N 12	NZ LYS 263 (A)	H-acceptor	2.83	−5.9
		6-ring	CD LYS 263 (A)	pi-H	3.69	−0.6
		6-ring	CD LYS 263 (A)	pi-H	4.65	−0.6
Cancer cells	7dae	N 18	N ASN 101 (A)	H-acceptor	3.44	−1.1
COVID 19	6W41	N 8	O ASN 450 (C)	H-donor	3.02	−2.1
		6-ring	CB SER 349 (C)	pi-H	4.22	−0.9

**Table 14:** Interaction energy values obtained from docking calculations of **Cd (II)-L**_**1**_ complex with different protein receptors

Type	Receptor	complex moiety	Receptor site	Interaction	Distance (A^ο^)	E (kcal/mol)
G^+^ Bacteria	3AHU	N 20	N PHE 62 (A)	H-acceptor	3.39	−2.4
	1GHP	N 29	OE1 GLN 237 (A)	H-donor	3.24	−3.0
	2ZIC	N 9	O GLY 227 (A)	H-donor	3.23	−2.8
		6-ring	ND2 ASN 222 (A)	pi-H	4.09	−0.7
G^−^ Bacteria	1NEK	N 29	O ALA 5 (D)	H-donor	2.94	−2
Fungi	5jpe	N 17	O ARG 386 (A)	H-donor	3.06	−10.2
		6-ring	NH2 ARG 386 (A)	pi-cation	4.19	−0.6
Cancer cells	7dae	N 8	OE1 GLU 71 (A)	H-donor	3.10	−5.1
		N 9	OD2 ASP 98 (A)	H-donor	3.47	−1.5
		N 17	OG1 THR 179 (A)	H-donor	3.32	−1.2
		O 33	N ASN 101 (A)	H-acceptor	2.94	−1.8
COVID 19	6W41	N 8	O ARG 355 (C)	H-donor	2.97	−2.5
		C 11	N ARG 355 (C)	H-acceptor	3.33	−6.8
		O 33	N ARG 357 (C)	H-acceptor	3.32	−1.9

## Conclusion

This study examines the tridentate Schiff base ligand (**L**_**1**_) and its ternary metal complexes through various analytical methods, including elemental analysis, molar conductance, mass spectroscopy, IR, and UV–visible spectroscopy. The findings from these analyses revealed the formation of octahedral-shaped complexes with Co(II), Cd(II), Mn(II), and Cu(II). The antimicrobial assessment displayed that the Cd-complex exhibited notably higher toxicity against bacterial and fungal species, rivaling most antibiotics, albeit falling short in potency compared to ampicillin in combating *Streptococcus mutans*. Furthermore, the (**L**_**1**_) Schiff base and its metal complexes, particularly Co(II), Cd(II), Mn(II), and Cu(II), exhibited remarkable inhibitory effects against *H. pylori*. The metal complexes of the **L**_**1**_ Schiff base, particularly Co(II), Cd(II), Mn(II), and Cu(II), demonstrated substantial activity against MCF-7 (*Breast carcinoma*).

## Data Availability

The datasets used and/or analyzed during the current study are available from the corresponding author upon reasonable request.

## Abbreviations

L_1_: Schiff Base Ligand (4-(2-((1E,2E)-1-(2-(p-tolyl)hydrazineylidene)propan-2-ylidene)hydrazineyl)quinazoline)
DFT: Density functional theory
DMF: N, N‐dimethylformamide
DMSO: Dimethyl sulfoxide
ICP: Inductively coupled plasma
FT-IR: Fourier-transform infrared spectroscopy
UV–Vis: Ultraviolet–Visible spectroscopy
NMR: Nuclear magnetic resonance
*H. pylori*: *Helicobacter pylori*
AMX: Amoxicillin
MTZ: Metronidazole
PDB: Protein data bank
LUMO: Lowest unoccupied molecular orbital
χ: Absolute electronegativity
σ: Absolute softness
S: Global softness
ΔNmax: Additional electronic charge
IC_50_: Inhibitory concentration 50%
L_2_: Glycine
TG: Thermogravimetric analysis
DTG: Differential thermogravimetric analysis
SRB: Sulforhodamine B
TCA: Trichloroacetic acid
RPMI: Roswell Park Memorial Institute medium
FBS: Fetal bovine serum
MBC: Minimal bactericidal concentration
MIC: Minimal inhibitory concentration
CLR: Clarithromycin
EDTA: Ethylenediaminetetraacetic acid
HOMO: Highest occupied molecular orbital
ΔE: Energy gap
η: Absolute hardness
Pi: Chemical potential
ω: Electrophilicity index
CFU: Colony-forming units
MCF-7: Breast carcinoma cell line
VERO cells: Normal cell line which is derived from the kidney of an African green monkey
